# Unassisted self-healing photocatalysts based on Le Chatelier’s principle

**DOI:** 10.1038/s42004-025-01500-7

**Published:** 2025-04-14

**Authors:** Aito Takeuchi, Yoshitaka Kumabe, Takashi Tachikawa

**Affiliations:** 1 Department of Chemistry, Graduate School of Science, Kobe University, 1-1 Rokkodai-cho, Kobe, 657-8501 Japan; 2 Molecular Photoscience Research Center, Kobe University, 1-1 Rokkodai-cho, Kobe, 657-8501 Japan

**Keywords:** Photocatalysis, Single-molecule fluorescence, Materials chemistry

## Abstract

Self-healing is a fundamental ability inherent in humans, plants, and other living organisms. To date, a variety of materials with self-healing properties have been developed. However, these materials usually require external inputs such as electric potentials or healing agents to initiate or promote self-healing reactions. Herein, we present a novel self-healing mechanism that operates without any external input, utilizing the dynamic equilibrium between the solid-state and dissolved materials. We employed organic–inorganic perovskites to validate our strategy. Single-particle spectroscopy and imaging demonstrated the spontaneous self-healing of perovskites after photodamage under dynamic equilibrium conditions. Furthermore, we found that perovskites can generate hydrogen in both healed and damaged states. Remarkably, the perovskites exhibited hydrogen generation over four cycles of photodamage and self-healing. The proposed concept and experimental results provide valuable insights for the development of energy conversion and storage systems with improved long-term durability.

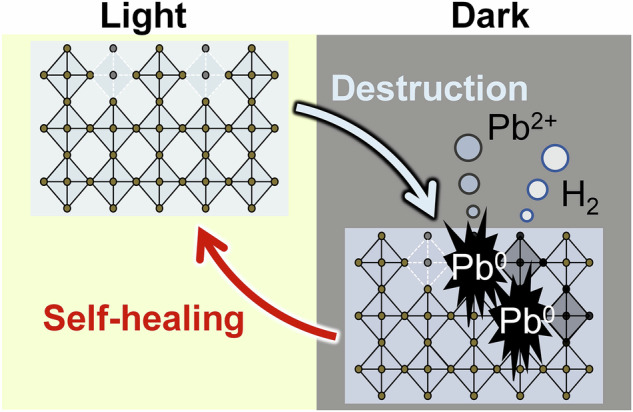

## Abstract (web summary)

Materials with self-healing properties are highly desirable for a wide range of applications, however most self-healing materials developed to date typically require external inputs to initiate or promote self-healing reactions. Here, self-healing perovskite-based photocatalysts that utilize the dynamic equilibrium between the solid-state and dissolved materials are found to heal without any external input.

## Introduction

**Fig. 1:**
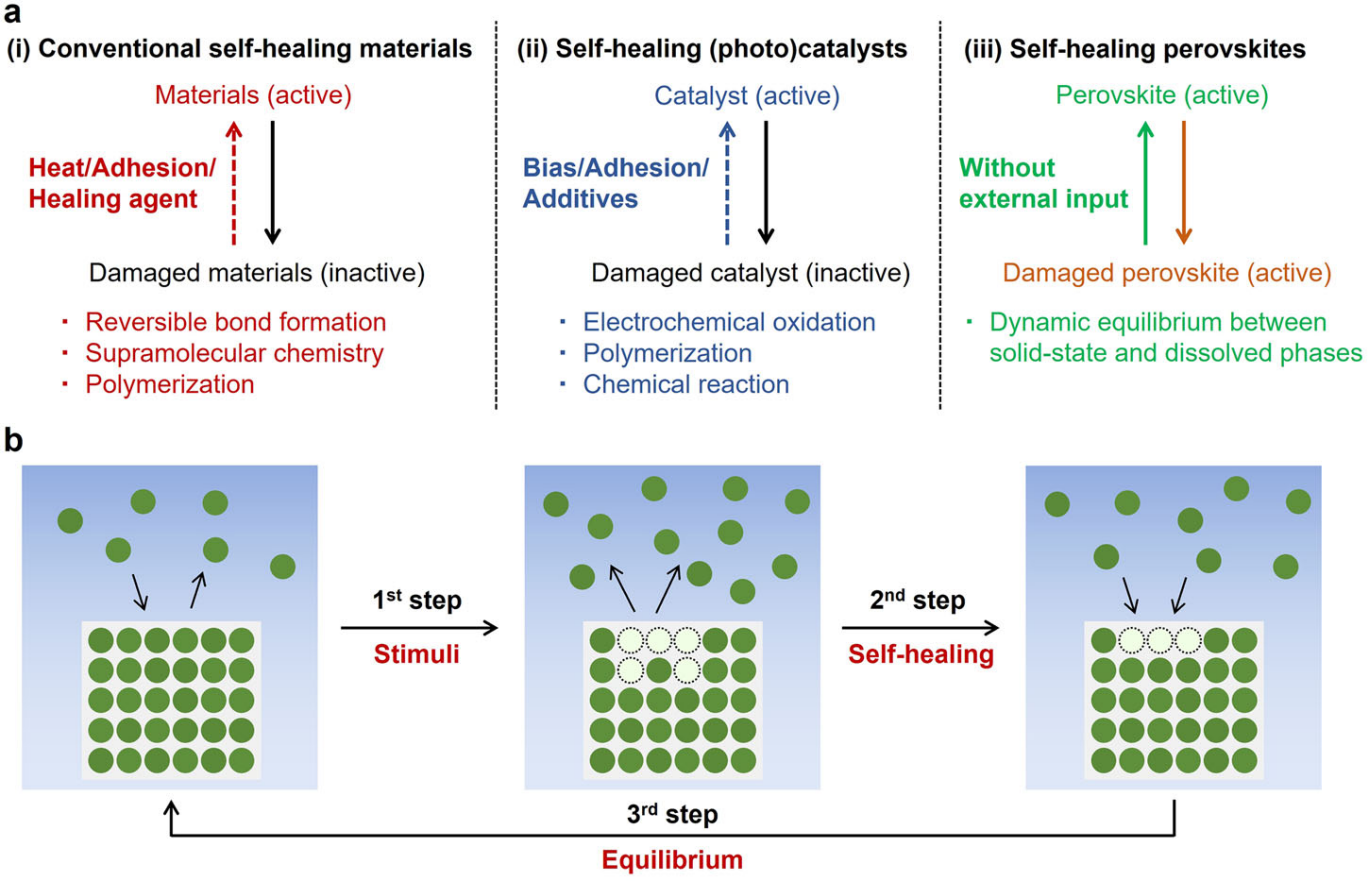
Overview of self-healing reactions based on Le Chatelier’s principle. **a** Comparison of self-healing mechanisms. (i) Conventional self-healing materials such as polymers, glasses, and metals. (ii) Self-healing (photo)catalysts such as photoelectrodes and air-reactive catalysts. (iii) Our newly developed self-healing system. In the system, self-healing reactions on the material occur spontaneously without external inputs. **b** Schematic illustration of self-healing mechanism based on Le Chatelier’s principle. When equilibrium is disturbed due to material damage (first step), the reaction system tends to shift to mitigate the disturbance (second step). Following the completion of the self-healing process, the reaction system returns to equilibrium (third step).

The ability to heal is crucial for living organisms. When skin is injured or a bone is fractured, the damaged biological tissues naturally repair themselves over time. Providing self-healing capabilities to other non-biological materials can significantly enhance their durability. For several decades, researchers have been actively investigating biomimetic self-healing materials, as conceptually summarized in Fig. 1a^1,2^. In conventional materials (Fig. 1a, left), self-repairing systems can be classified into those with intrinsic and extrinsic mechanisms. The intrinsic mechanism utilizes the atoms and bonds of the material itself, which is mainly found in the self-healing of polymers^3–5^. The extrinsic mechanism employs healing agents, such as capsules or nanoparticles^6,7^. Overall, these intrinsic and extrinsic self-healing materials require external inputs such as heat, adhesives, or healing agents to facilitate the processes.

Self-healing (photo)catalysts have also been investigated (Fig. 1a, middle)^8–10^. Nocera et al. reported oxygen-generating self-healing photoelectrodes that required an appropriate applied bias^11–13^. In this system, Co^4+^ ions loaded onto the electrode served as catalytic sites. As oxygen generation proceeds via water oxidation, Co^4+^ is reduced, leading to the dissociation of Co cations from the electrode and a decrease in catalytic durability. When the applied potential was sufficient to oxidize the Co cations, the redeposition of Co cations proceeded simultaneously, allowing for further photocatalytic reactions. Other metal-based self-healing photoelectrodes have been reported^14^; however, most of these catalysts cannot self-heal without an external input (e.g., an electric potential) in catalytically active environments.

Herein, we propose a newly designed unassisted self-healing mechanism based on dynamic equilibrium (Fig. 1a, right). When materials in this dynamic equilibrium state are damaged, the equilibrium shifts towards self-healing reactions because of Le Chatelier’s principle. This principle states that if a system in equilibrium is disturbed, the system adjusts to minimize the disturbance. Figure 1b illustrates a reaction scheme facilitated by Le Chatelier’s principle. In the first step, the original materials reach a dynamic equilibrium between the solid state and their dissolved form. When the materials are damaged or several components are eluted, this dynamic equilibrium is disturbed by the increased concentration of chemicals in the solution. According to Le Chatelier’s principle, the equilibrium shifts towards reactions that reduce the concentration of chemicals in the solution (second step). Finally, self-healing of the materials is completed (third step). This self-healing reaction operates without the need for external assistance, and the key factor is maintaining the reaction system in a dynamic equilibrium state. The degradation and self-healing of materials are reversible reactions that allow continued use.

In this study, organic–inorganic perovskites are employed as exemplary materials to demonstrate our concept. Halide perovskites are promising light-harvesting and light-emitting materials for applications such as solar cells, light-emitting diodes (LEDs), and lasers owing to their favorable optical properties^15–20^. However, these perovskites are inherently unstable under humid conditions. Nonetheless, recent studies have demonstrated that perovskites can exist stably in saturated aqueous solutions, reaching a dynamic equilibrium between the solid-state and dissolved phases^21^. Under these conditions, they can act as photocatalysts for hydrogen generation under visible light irradiation^22,23^. We evaluated the self-healing behavior of perovskites in dynamic equilibrium at the single-particle level using a fluorescence microscope. We demonstrated that photodamaged crystals in the system possess self-healing abilities without requiring any external input. Interestingly, even in their damaged state, perovskites continue to produce hydrogen gas in aqueous solution, indicating that they can function as (photo)catalysts in both healed and damaged states. These findings suggest a possible strategy for the development of sustainable systems.

## Results and discussion

### Single-particle observations of crystal destruction and self-healing

Halide perovskites are unstable in the presence of humidity^24,25^, oxygen^26,27^, and heat^28,29^. However, when they are in a dynamic equilibrium state, the effects of these stimuli may be mitigated because the saturated solution acts as a passivating medium. Therefore, we employed single-particle fluorescence microscopy to directly monitor structural changes in individual perovskite crystals in response to light irradiation. This technique allows the evaluation of nanoscopic heterogeneities in chemical reactions at solid-liquid interfaces, which are typically obscured in ensemble-averaging bulk measurements^30–32^.

**Fig. 2:**
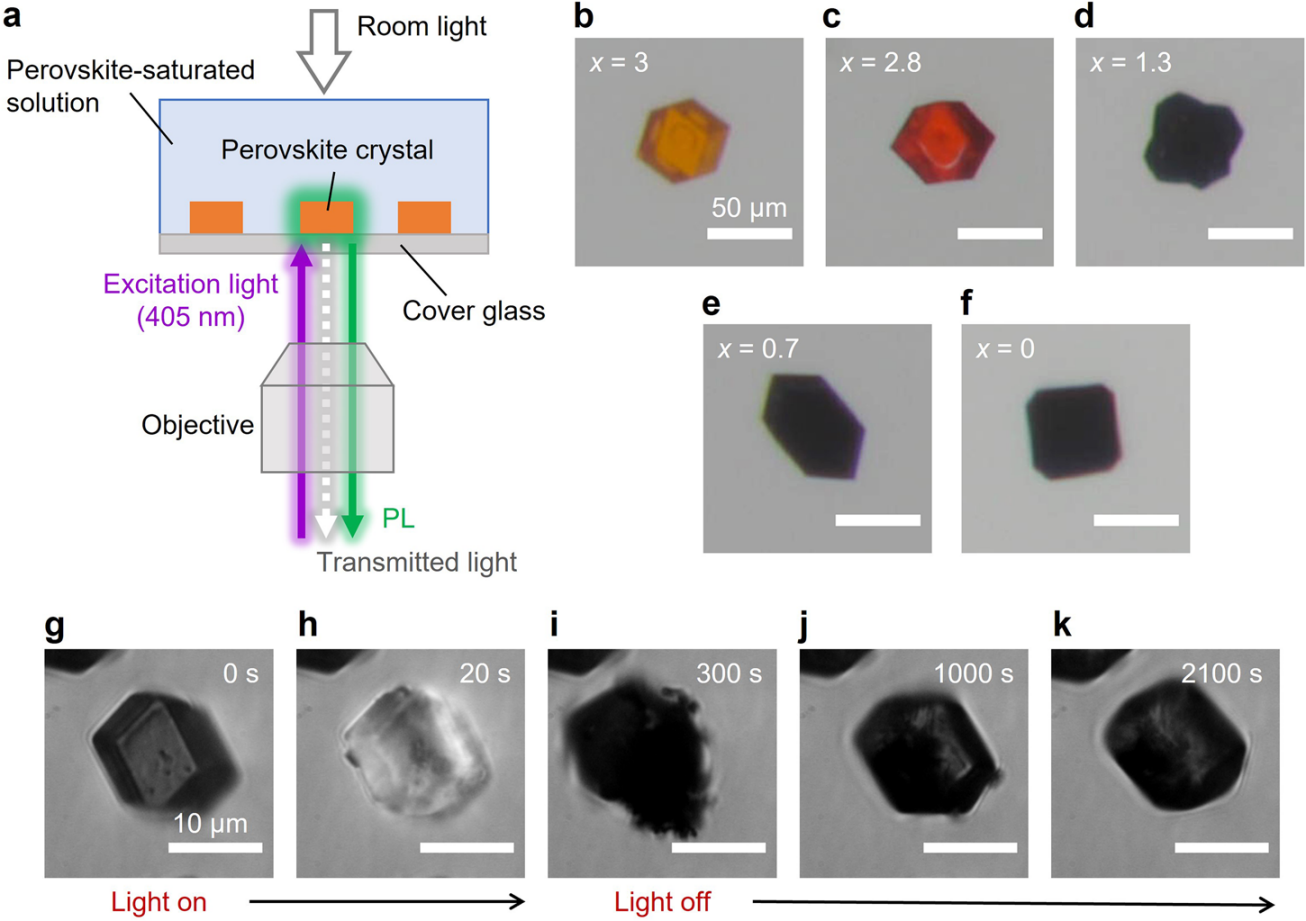
Single-particle observations using microscopic techniques. **a** Experimental setup for single-particle measurements. The experimental setup is based on a wide-field fluorescence microscope system. Both PL and transmitted light were captured using the same objective lens. **b–f** Optical images of mixed-halide MAPbBr_*x*_I_3−*x*_ perovskites with various *x*-values in aqueous solution. **g–k** Optical transmission images in aqueous solution. The inset labels indicate the time after the start of photoirradiation. Excitation was provided for the first 300 s, after which photoirradiation was stopped. A 405-nm CW laser (ca. 780 mW·cm^−2^ at the sample surface) was used as the excitation source.

Figure 2a depicts the experimental setup for single-particle observation using a home-built inverted fluorescence microscope system^33,34^. MAPbBr_*x*_I_3−*x*_ microcrystals (where MA^+^ = CH_3_NH_3_^+^) were spin-coated onto a cleaned cover glass to serve as seed crystals. A supersaturated perovskite solution was prepared by heating a suspension containing the target crystals at 80 °C, which was immediately dropped onto the substrate. As the temperature of the solution decreased, the perovskite crystals reprecipitated owing to the reduced solubility. Few studies have been conducted on the synthesis of perovskites in aqueous media^35–37^. Therefore, we investigated the correlation between the halide composition of the prepared solution and that of the resulting crystals. Interestingly, we found that iodide anions tended to be incorporated into the crystals more readily than bromide anions (Supplementary Fig. 1a). A better correlation was observed between the iodide/lead ratios of the prepared solution and those of crystals (Supplementary Fig. 1b). A discussion of this tendency is provided in Supplementary Note 1. After allowing the crystals to grow sufficiently, we initiated imaging of the crystal morphology and spectroscopic measurements (Supplementary Fig. 1c). Figure 2b–f illustrates optical images of the obtained MAPbBr_*x*_I_3−*x*_ crystals under dynamic equilibrium. The halide compositions were determined based on data from X-ray diffraction (XRD)^38^ (Supplementary Fig. 1d). Photoluminescence (PL) imaging of individual perovskite crystals with various halide compositions revealed that the size of the MAPbBr_3_ and MAPbI_3_ crystals remained unchanged, or slightly decreased, or increased during 405-nm continuous wave (CW) laser irradiation (Supplementary Figs. 2 and 3). However, in the case of the mixed-halide MAPbBr_2.8_I_0.2_ crystals, the crystal morphology changed drastically when the excitation density was at least 780 mW·cm^−2^ or higher at the sample surface (Fig. 2g–i and Supplementary Movie 1), and we refer to this morphological change as crystal destruction. When the damaged crystals in the aqueous solution were left in the dark after crystal destruction, self-healing behavior was observed (Fig. 2j, k and Supplementary Movie 1). This finding indicates that the proposed self-healing mechanism based on dynamic equilibrium applies to perovskites.

### Mechanisms of crystal destruction and self-healing

**Fig. 3:**
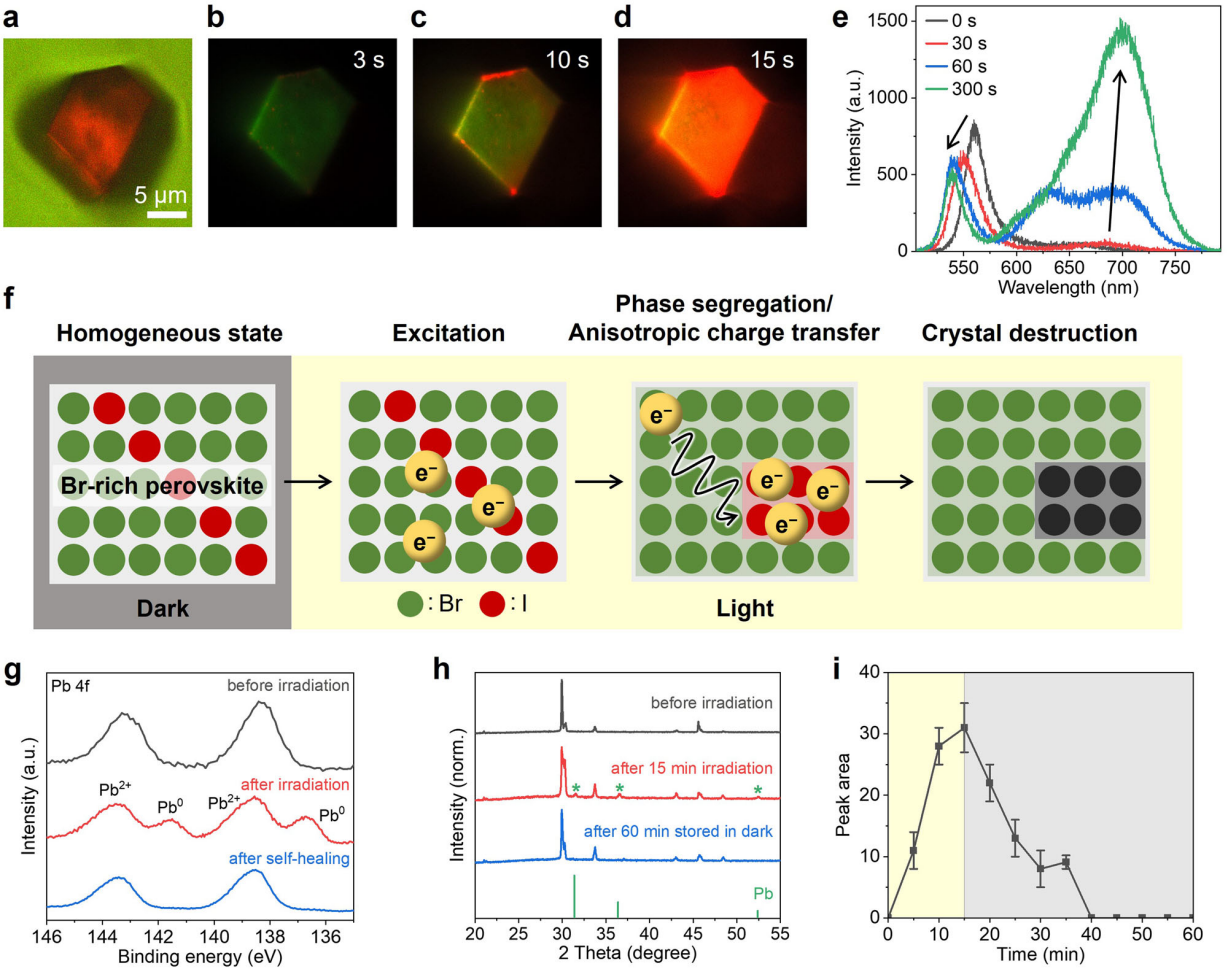
Damaging and self-healing reactions of perovskites. Optical transmission and PL images of MAPbBr_2.8_I_0.2_ in aqueous solution before (**a**) and during photoirradiation (**b–d**). The inset labels show the time elapsed from the start of light irradiation. A 405-nm CW laser (ca. 1.21 W·cm^−2^ at the sample surface) was used as the excitation source. **e** Spectral changes of the MAPbBr_2.8_I_0.2_ crystal in aqueous solution under irradiation. A 405-nm pulsed laser (ca. 8×10^−17^ J·pulse^−1^) was used as excitation source. **f** Schematic illustration of the crystal destruction mechanism induced by halide phase segregation. **g** Pb 4 f XPS spectra of MAPbBr_2.8_I_0.2_ before and after irradiation, and after self-healing. A 405-nm LED (ca. 350 mW·cm^−2^) was used as excitation source. **h** XRD patterns of MAPbBr_2.8_I_0.2_ in aqueous solution before and after irradiation. A 405-nm LED (ca. 350 mW·cm^−2^) was used as excitation source. Asterisks indicate characteristic peaks of metallic Pb (PDF card: 00-004-0686). **i** Temporal changes in the Pb^0^ peak area under irradiation (yellow-shaded region) and after stopping irradiation (gray-shaded region). The error bars represent the standard deviation.

Next, we investigated the mechanisms underlying the observed crystal destruction and self-healing behaviors. To determine why only the mixed-halide MAPbBr_2.8_I_0.2_ crystals were destroyed, we conducted color imaging of the perovskites under laser irradiation. Figure 3a, b–d displays the optical transmission image of a MAPbBr_2.8_I_0.2_ crystal before laser irradiation and the PL images of MAPbBr_2.8_I_0.2_ under 405-nm CW laser irradiation, respectively. Initially, the MAPbBr_2.8_I_0.2_ crystal exhibited a weak green emission. As the laser irradiation time increased, the edges of the crystal began to exhibit red emission. Finally, a bright red emission was observed from the entire crystal. Spectral measurements of a specific region (approximately 2 µm^2^ in area) of the crystal revealed that MAPbBr_2.8_I_0.2_ initially exhibited PL at approximately 560 nm. As the irradiation time increased, however, this peak exhibited a slight blue shift and a broad emission band appeared at longer wavelengths. The intensity of the latter component increased and red-shifted, eventually reaching a maximum at approximately 700 nm (Fig. 3e and Supplementary Fig. 4). These characteristic PL changes were observed repeatedly (Supplementary Fig. 5) and were attributed to light-induced phase segregation^39^. To explain the origin of phase segregation, several models have been proposed^40^, including those based on the miscibility gap due to thermodynamically unstable mixed-halide states, polaron-induced lattice strains that increase the mixing enthalpy of halide anions, and electric field-driven ion migration induced by the trapping of charge carriers at defects. In any cases, based on the band structures of perovskites, photogenerated charge carriers migrate from bromide-rich domains to iodide-rich domains with a narrower band gap, leading to the appearance of red-shifted PL (Supplementary Fig. 6). Similar PL features related to phase segregation have been reported elsewhere^41–43^; however, these studies did not involve conditions of aqueous dynamic equilibrium. We also conducted the same measurements on MAPbBr_0.3_I_2.7_ and MAPbBr_1.3_I_1.7_ (Supplementary Fig. 7). MAPbBr_1.3_I_1.7_ exhibited a similar red-shifted PL, followed by crystal destruction and self-healing reactions. In contrast, MAPbBr_0.3_I_2.7_ did not show such behavior and retained their original morphology without any damage. Furthermore, we examined whether phase segregation induces the destruction of crystals with extreme halide compositions. In this experiment, a supersaturated HI (HBr) solution was dropped onto a MAPbBr_3_ (MAPbI_3_)-coated cover glass. Hereafter, we refer to each resulting crystal as MAPbI_3_ (MAPbBr_3_) with a small amount of bromide (iodide) based on the PL peak at approximately 760 nm (540 nm) (Supplementary Figs. 8a and 9a). Upon exposure to light, the morphology of MAPbI_3_ with a small amount of bromide remained unchanged (Supplementary Fig. 8b, c), whereas the MAPbBr_3_ crystals with a small amount of iodide were destroyed in a manner similar to that of MAPbBr_2.8_I_0.2_ (Supplementary Fig. 9c, d). As illustrated in Supplementary Fig. 9b, the PL spectrum of MAPbBr_3_ with a small amount of iodide not only exhibits dominant green PL at approximately 540 nm but also weak red PL at approximately 700 nm, indicating that light-induced phase segregation occurred even with an extremely small amount of iodide anions. These results indicate phase segregation induces the crystal destruction. Therefore, self-healing effect is not exclusive to the MAPbBr_2.8_I_0.2_, it can be applicable to other composition perovskites.

Based on the aforementioned results, we conclude that phase segregation is essential for crystal destruction (Fig. 3f). Prior to light irradiation, halide anions are uniformly distributed within the crystal. Upon photoexcitation of the mixed-halide perovskites, phase segregation mainly occurs in the regions near the surface, considering the limited penetration depth ( ~ 120 nm) of the excitation light^44^. Holes are trapped by chemical species such as halide anions in solution^23^, leading to the accumulation of excess electrons in the localized iodide-rich regions. The accumulated electrons reduce the perovskites, leading to crystal destruction and morphological changes. Such significant crystal destruction was not observed in air, where no halide anions were present. The MAPbI_3_ with a small amount of bromide did not decompose because the charge carriers remained within the iodide-rich regions that constitute most of the crystal (Supplementary Fig. 10). This proposed mechanism is supported by simultaneous imaging of the PL color and crystal morphology of MAPbBr_2.8_I_0.2_ crystals (Supplementary Fig. 11). Upon laser irradiation, MAPbBr_2.8_I_0.2_ emitted red PL, attributed to the iodide-rich domains formed by phase segregation. Once crystal destruction occurred, the regions near the damaged areas began to emit green PL, corresponding to bromide-rich domains, whereas red PL persisted in areas distant from the damage.

To assess the electronic states of MAPbBr_2.8_I_0.2_, we performed X-ray photoelectron spectroscopy (XPS). The Pb 4 f XPS spectra in Fig. 3g exhibit only Pb^2+^ peaks before photoirradiation (black line), whereas Pb^0^ peaks appeared after photoirradiation (red line). The formation and disappearance of metallic Pb were further confirmed by in-situ XRD measurements and scanning electron microscopy-energy dispersive X-ray spectrometry (SEM-EDS) (Fig. 3h and Supplementary Fig. 12). In the XRD pattern, Pb^0^ peaks were clearly observed, and no broad amorphous peaks appeared after photoirradiation. Therefore, we consider that most of the Pb^0^ is in the crystalline phase. Figure 3i illustrates the temporal change in the Pb peak area. The perovskites were irradiated for 15 min, after which the light was turned off. With increasing photoirradiation time, the Pb peak area also increases. Photodegradation resulting from the generation of Pb^0^ in mixed-halide perovskites via phase segregation has been reported previously^45,46^, suggesting that our proposed mechanism is plausible. Such crystal destruction and self-healing reactions were further investigated using the diffuse reflectance spectra of MAPbBr_2.8_I_0.2_ (Supplementary Fig. 13). After photoirradiation, a broad band, possibly attributed to the absorption of photogenerated metallic Pb, was observed. This band nearly disappeared when the sample was kept in the dark.

Regarding the self-healing behavior in aqueous solution, we consider the dynamic equilibrium of perovskites as follows^21^:

1 $${{{\rm{MAPb}}}}{{{{\rm{Br}}}}}_{x}{{{{\rm{I}}}}}_{3-x}({{{\rm{s}}}})\rightleftharpoons {{{{\rm{MA}}}}}^{+}+{\left[{{{\rm{Pb}}}}{{{{\rm{Br}}}}}_{x}{{{{\rm{I}}}}}_{3-x}\right]}^{-}$$

In aqueous solution, various chemicals such as bromide anions, iodide anions, and phosphinic acids are present, with the latter being particularly important for reducing oxidative species such as triiodide anions. The standard redox potentials of the relevant reactions and the band structures of MAPbX_3_ (X = Br, I) are summarized in Supplementary Fig. 14. The Pb^0^ generated in the perovskite-saturated aqueous solution is oxidized to Pb^2+^ according to the following reaction:

2 $${{{{\rm{Pb}}}}}^{0}+2{{{{\rm{H}}}}}^{+}\to {{{{\rm{Pb}}}}}^{2+}+{{{{\rm{H}}}}}_{2}$$

Protons are the dominant electron acceptors in aqueous solution because of the presence of phosphinic acid. The Gibbs free energy change (Δ*G*) of Eq. (2) is determined by the following equation:

3 $$\Delta G=-{nF}\Delta E$$

where *n* is the number of electrons involved in the reaction, *F* is Faraday’s constant, and Δ*E* is the potential difference between the related half-reactions. The calculated Δ*G* is −24.3 kJ·mol^−1^^47^, indicating that the oxidation of metallic Pb^0^ to Pb^2+^ proceeds spontaneously when Pb^0^ is generated by the destruction of perovskites. Due to the high concentration of halide anions in the aqueous solution, lead complexes form^48^.

4 $${{{{\rm{Pb}}}}}^{2+}+x{{{{\rm{Br}}}}}^{-}+\left(3-x\right){{{{\rm{I}}}}}^{-}\to {\left[{{{\rm{Pb}}}}{{{{\rm{Br}}}}}_{x}{{{{\rm{I}}}}}_{3-x}\right]}^{-}$$

Based on Le Chatelier’s principle, the formation of [PbBr_*x*_I_3−*x*_]^−^ causes a shift in the dynamic equilibrium, resulting in the production of MAPbBr_*x*_I_3−*x*_(s), as expressed in Eq. (1). Consequently, the perovskite material self-heals. This self-healing behavior was also observed using in situ XRD measurements and diffuse-reflectance spectra. Upon stopping the light irradiation, the peak area associated with Pb^0^ gradually decreased and eventually disappeared completely (Fig. 3i). After leaving damaged perovskites in the solution under dark condition, absorption of photons with lower than bandgap energy was drastically decreased, indicating that self-healing reactions are proceeded (Supplementary Fig. 13). Supplementary Fig. 15 shows PL spectra before photodamaging, after photodamaging, and after self-healing reactions. In this figure, the PL wavelength, which reflects the halide composition of perovskites, remained unchanged after the self-healing process. Hence, the photodamaging and self-healing processes do not affect the Br/I ratio of the crystal. Based on the formation of MAPbBr_3_ from Pb powder in an aqueous HBr solution containing MABr as the organic cation source (Supplementary Fig. 16), the self-healing formation of mixed-halide perovskites is thermodynamically and kinetically feasible. This self-healing behavior, achieved without external stimuli, has significant implications for the development of novel catalysts that utilize a dynamic equilibrium reaction system.

### Photocatalytic hydrogen evolution

**Fig. 4:**
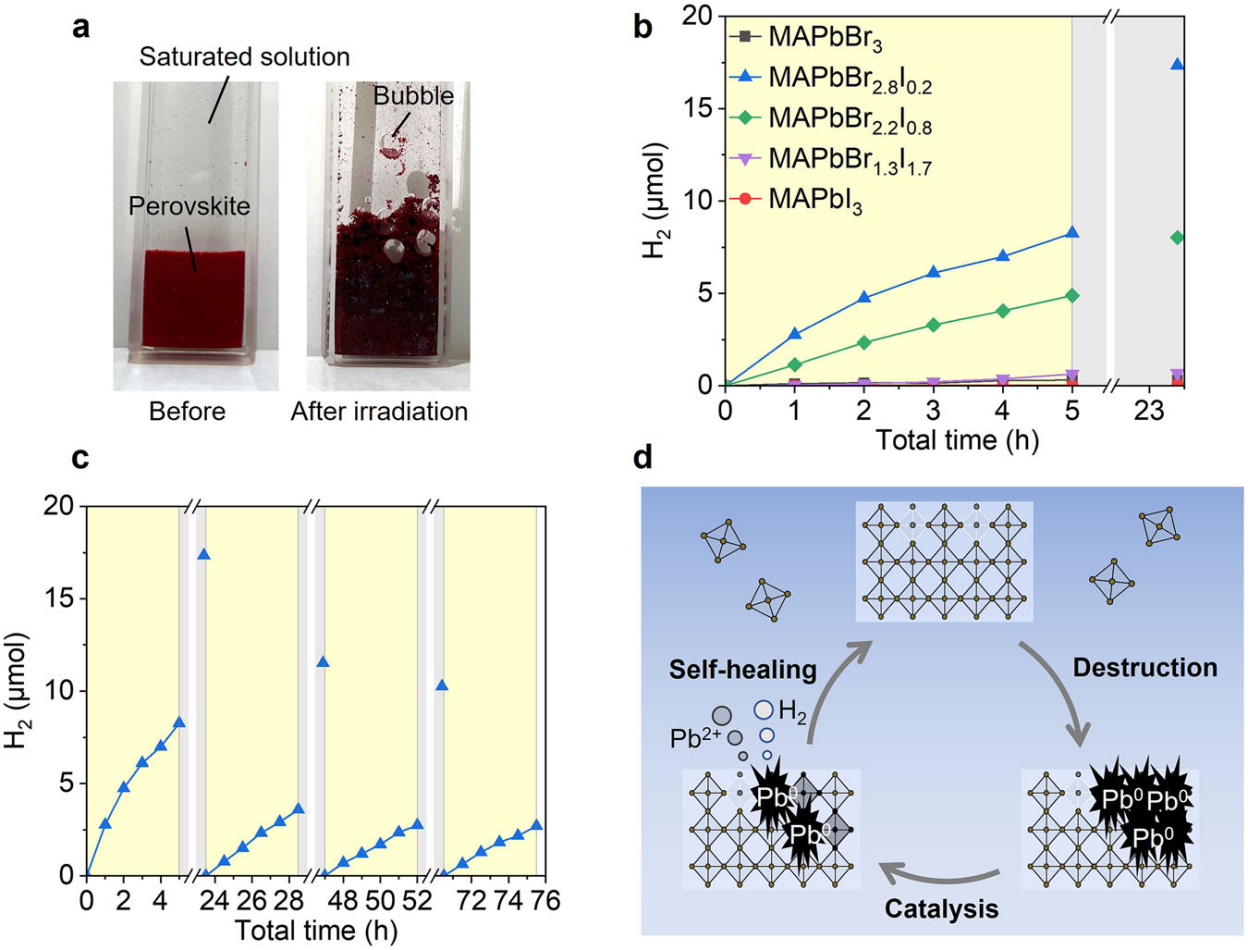
(Photo)catalytic hydrogen-production activity of perovskites under visible-light irradiation in aqueous solution. **a** Optical images of the MAPbBr_2.8_I_0.2_ powder in saturated aqueous solution (left) before and (right) after 470-nm LED light irradiation (ca. 125 mW·cm^−2^) for 24 h. **b** Total amount of hydrogen generated by perovskites, indicating the catalytic activity of MAPbBr_3_ (black line and symbols), MAPbI_3_ (red line and symbols), MAPbBr_1.3_I_1.7_ (purple line and symbols), MAPbBr_2.2_I_0.8_ (green line and symbols) and MAPbBr_2.8_I_0.2_ (blue line and symbols). The yellow-shaded region indicates the period of light irradiation, while the gray-shaded region indicates the period of darkness. **c** The hydrogen production of MAPbBr_2.8_I_0.2_ during intermittent irradiation. **d** Schematic illustration of degradation and self-healing reactions in aqueous solution under dynamic equilibrium. The self-healing reaction occurs spontaneously once the perovskites are damaged, and this cycle can continue repeatedly.

Upon light exposure, the MAPbBr_2.8_I_0.2_ powder in a saturated aqueous solution exhibited a color change from reddish brown to black, due to the formation of metallic Pb (Fig. 4a and Supplementary Fig. 13)^49^. Moreover, numerous bubbles formed on the particles. As illustrated in Supplementary Fig. 14, the conduction band minimum of MAPbX_3_ is more negative than the potential for proton reduction, enabling photocatalytic hydrogen generation as described in the following equations^23,36,50^:

5 $${{{{\rm{MAPbBr}}}}}_{x}{{{{\rm{I}}}}}_{3-x}+h\nu \to {{{\rm{MAPb}}}}{{{{\rm{Br}}}}}_{x}{{{{\rm{I}}}}}_{3-x}({{{{\rm{h}}}}}^{+})+{{{\rm{M}}}}{{{{\rm{APbBr}}}}}_{x}{{{{\rm{I}}}}}_{3-x}({{{{\rm{e}}}}}^{-})$$

6 $${{{\rm{MAPb}}}}{{{{\rm{Br}}}}}_{x}{{{{\rm{I}}}}}_{3-x}(2{{{{\rm{h}}}}}^{+})+3{{{{\rm{I}}}}}^{-}\to {{{\rm{MAPb}}}}{{{{\rm{Br}}}}}_{x}{{{{\rm{I}}}}}_{3-x}+{{{{\rm{I}}}}}_{3}^{-}$$

7 $${{{\rm{MAPb}}}}{{{{\rm{Br}}}}}_{x}{{{{\rm{I}}}}}_{3-x}(2{{{{\rm{h}}}}}^{+})+2{{{{\rm{Br}}}}}^{-}\to {{{\rm{MAPb}}}}{{{{\rm{Br}}}}}_{x}{{{{\rm{I}}}}}_{3-x}+{{{{\rm{Br}}}}}_{2}$$

8 $${{{\rm{MAPbBr}}}}x{{{{\rm{I}}}}}_{3-x}({2{{{\rm{e}}}}}^{-})+2{{{\rm{H}}}}^+ \to {{{\rm{MA}}}}{{{{\rm{PbBr}}}}}_{x}{{{{\rm{I}}}}}_{3-x}+{{{{\rm{H}}}}}_{2}$$

In addition, the photodamaging reaction occurred as follows:

9 $${{{\rm{MAPb}}}}{{{{\rm{Br}}}}}_{x}{{{{\rm{I}}}}}_{3-x}\left(2{{{{\rm{e}}}}}^{-}\right)\to {{{{\rm{MA}}}}}^{+}+{{{{\rm{Pb}}}}}^{0}+{x{{{\rm{Br}}}}}^{-}+(3-x){{{{\rm{I}}}}}^{-}$$

In the present dynamic equilibrium system, the self-healing reactions include hydrogen generation, as expressed in Eq. (2). Generally, photocatalysts are only active under photoirradiation. However, if our proposed mechanism holds true, perovskites can produce hydrogen not only when photoexcited but also under dark conditions. To test this hypothesis, we evaluated the photocatalytic hydrogen production activity using gas chromatography. Figure 4b compares the amounts of hydrogen gas produced during 5 h of visible light-driven HX splitting and 18.5 h of storage in the dark. In these experiments, the perovskites were able to produce hydrogen for 5 h, indicating that they can stably exist in the solution without complete crystal destruction for a longer duration compared to microscopic measurements. This is likely due to the difference in excitation power densities at the sample surface under each condition: ca. 780 mW·cm^−2^ for microscopic measurements (Fig. 2) and ca. 125 mW·cm^−2^ for photocatalytic measurements (Fig. 4). The photocatalytic activity of mixed-halide perovskites is significantly higher than those of the single-halide perovskites, MAPbBr_3_ and MAPbI_3_. One possible reason for the higher photocatalytic activity of the mixed-halide perovskites is light-induced phase segregation. During this process, iodide-rich domains are formed near the surface, enabling efficient charge transport to these domains and facilitating photocatalytic reactions^36^. This behavior explains how the original (undamaged) perovskites are effective photocatalysts. Hydrogen generation from the pre-irradiated samples held in the dark suggests that the damaged perovskites remained active during the self-healing reactions. As shown in Supplementary Fig. 17, the hydrogen generation rates under light are gradually decreased as light irradiation time increased, indicating that photocatalytic hydrogen evolution and self-healing-induced hydrogen evolution (Eq. (2)) occurred simultaneously. Another photocatalytic activity test supports our hypothesis. After initiating the photocatalytic reaction, we intermittently stopped the light irradiation. As illustrated in Supplementary Fig. 18, the system continued to exhibit stable hydrogen generation even in the dark. In our tests, the amount of Pb^2+^ in the MAPbBr_2.8_I_0.2_ crystals was approximately 20.5 µmol. However, the perovskites are not completely reduced to Pb^0^, as evidenced by their continued PL (Supplementary Fig. 11). This suggests that they retain part of their perovskite structure even after crystal destruction. Therefore, the actual amount of photogenerated Pb^0^ should be less than 20 µmol. Nonetheless, the total amount of hydrogen produced under dark conditions reached approximately 24.5 µmol (9.08, 7.98, and 7.51 for the first, second, and third cycles, respectively, Fig. 4c), again highlighting the self-healing capability of MAPbBr_2.8_I_0.2_ as a photocatalyst. The estimation of the potential lifetime of the photocatalytic reaction system, based on the amount of chemicals in the solution, is discussed in Supplementary Note 2. The hydrogen production activity of mixed-halide perovskites tends to decrease as the ratio of iodide anions in the crystals increases (Fig. 4b). This may be because the increased iodide content leads to a larger area of phase-segregated iodide-rich regions. Consequently, less efficient electron transfer occurs compared to MAPbBr_2.8_I_0.2_, resulting in lower hydrogen production activity and reduced susceptibility to destruction. This explanation is supported by microscopic observations (Supplementary Figs. 7–9).

However, with an increasing number of cycles, their photocatalytic activity gradually decreased. This could be attributed to insufficient self-healing processes. Supplementary Fig. 19 shows SEM-EDS images of MAPbBr_2.8_I_0.2_ prepared under various conditions. Before irradiation, each component—Br, I, and Pb—was homogeneously distributed across the entire crystals. After photoirradiation, I-rich and Pb-rich regions, corresponding to phase-segregated domains and photoreduced metallic Pb, respectively, were observed. Even after being left in their saturated solution, these I-rich and Pb-rich regions remained, suggesting that self-healing reactions were not completed within the experimental timescale. Furthermore, the solution may become supersaturated during irradiation due to the decomposition of crystals and the dissolution of their components.

Figure 4d summarizes the overall reaction scheme. Initially, perovskites stably exist in an aqueous solution by achieving dynamic equilibrium at the solid liquid interfaces. Upon photoexcitation, the accumulated electrons reduce Pb^2+^ in the perovskites to Pb^0^, resulting in significant morphological changes (Fig. 2i). After stopping light irradiation, self-healing reactions occur along with hydrogen generation. The reconstructed (self-healed) perovskites again function as photocatalysts for continued use.

Supplementary Fig. 20 illustrates the cycle of deciduous trees^51^. When temperatures cool, dormancy is induced, leading to the transfer of nitrogen (N), an energy source for plants, from leaves to stems. Subsequently, the leaves are shed. The stored nitrogen is then used during bud break in the next cycle to produce new growth. The self-healing mechanism of perovskites demonstrated in this study is analogous to this process. In the damaged state, akin to the dormant state in plants, perovskites store energy in the form of charges in the metallic Pb^0^ regions. The self-healing reaction, which is comparable to bud break and leaf growth in plants, is driven by the utilization of both stored chemical energy and thermal energy. The similarity between the processes in perovskites and plants suggests that biomimicry is a powerful strategy for achieving efficient energy utilization.

## Conclusions

In summary, we developed novel self-healing systems incorporating mixed-halide perovskites, based on Le Chatelier’s principle. Unlike other self-healing materials or systems, the proposed mechanism does not require external input to induce a self-healing reaction under dynamic equilibrium conditions. We further demonstrated that this self-healing system applies to photocatalytic hydrogen generation. Even if the self-healing perovskites are damaged by prolonged light exposure, they can stably produce hydrogen in the dark. This self-healing capability is particularly beneficial for the practical application of photocatalytic systems, as it accounts for the day-night cycle on our planet. During the day, the materials function as photocatalysts, and at night, they heal. The dynamic transition between the solid state and the dissolved state is fundamental to our self-healing mechanism, thereby making it applicable to a wide range of materials, including those based on non-covalent bonds (e.g., metal-organic frameworks and organic crystals). We believe that our proposed mechanism could serve as a design principle for innovative and sustainable self-healing materials and systems for photocatalysis and other applications.

## Methods

### Chemicals

Hydriodic acid (HI) (Sigma-Aldrich; 57 wt% in water, 99.99% trace metal basis), lead (Pb) (Sigma-Aldrich; 99.5%), methylamine hydroiodide (MAI) (TCI; low water content. >99.0%), lead(II) iodide (PbI_2_) (TCI; 99.99%, trace metal basis, >98.0%), methylamine hydrobromide (MABr) (TCI; low water content, >98.0%), lead(II) bromide (PbBr_2_) (TCI; >98.0%), γ-butyrolactone (TCI; >99.0%), phosphinic acid solution (FUJIFILM Wako Chemicals; 50 wt% in water), hydrobromic acid (HBr) (FUJIFILM Wako Chemicals; 47.0–49.0 wt% in water), toluene (FUJIFILM Wako Chemicals; super dehydrated, >99.5%), N,N-dimethylformamide (DMF) (FUJIFILM Wako Chemicals; super dehydrated, >99.5%), 2-propanol (FUJIFILM Wako Chemicals; super dehydrated, >99.7%), were used without further purification.

### Synthesis of MAPbX_3_ microcrystals by using an aqueous solution and preparation of MAPbX_3_-saturated HX solution (X = Br, I)

Equimolar amounts of MAX and PbX_2_ were added to the same amount of HX/H_3_PO_2_ solution (HX : H_3_PO_2_ = 4 : 1, v/v), respectively. These solutions were sonicated, and then MAX solution was added to the PbX_2_ solution to initiate the crystallization of MAPbX_3_. The mixed solution was heated in an oil bath at 100 °C for 1 h. After heating, the solution was allowed to cool to room temperature. The supernatant solution was collected and stored as MAPbX_3_-saturated HX solution for further experiments. The obtained crystals were washed several times with toluene and then dried in a vacuum dryer at 45 °C.

### Synthesis of MAPbBr_*x*_I_3−*x*_ microcrystals by using an aqueous solution and preparation of MAPbBr_*x*_I_3−*x*_-saturated HBr/HI solution

MAPbBr_3_ synthesized via aqueous media was used as seed crystals. A certain amount of MAPbBr_3_-saturated HBr solution and MAPbI_3_-saturated HI solution were added to MAPbBr_3_ crystals to mix halide anions. The mixed solution was heated in an oil bath at 100 °C for 30 min. After heating, the solution was allowed to cool to room temperature. The supernatant solution was used as MAPbBr_*x*_I_3−*x*_-saturated HBr/HI solution for further experiments. The obtained crystals were washed several times with toluene and then dried in a vacuum dryer at 45 °C.

### Synthesis of MAPbBr_3_ microcrystals by using an organic solvent

81.8 mmol of MABr and PbBr_2_ were added to 2 mL of DMF and the solution was sonicated to completely dissolve the precursors. 4 mL of toluene was slowly added dropwise to the precursor solution. The obtained crystals were separated by centrifugation and washed several times with toluene.

### Synthesis of MAPbI_3_ microcrystals by using an organic solvent

0.25 mmol of MAI and PbI_2_ were each added to 1 mL of 2-propanol, and both solutions were sonicated. After sonication, the PbI_2_ solution was added dropwise to the MAI solution. The obtained crystals were separated by centrifugation and washed several times with toluene.

### Synthesis of MAPbBr_*x*_I_3−*x*_ microcrystals by using an organic solvent

To prepare the bromide-precursor solution, 81.8 mmol of MABr and PbBr_2_ were added to 8 mL of γ-butyrolactone. Similarly, 81.8 mmol of MAI and PbI_2_ were added to 3 mL of γ-butyrolactone to prepare the iodide-precursor solution. Each precursor solution was sonicated to completely dissolve the chemicals. A certain amount of bromide-precursor and iodide-precursor solutions were then mixed, ensuring the total volume of the mixed solution was 2 mL. Subsequently, 4 mL of toluene was slowly added dropwise to the precursor solution. The obtained crystals were separated by centrifugation and washed several times with toluene.

### Characterizations

X-ray diffraction (XRD) patterns were measured on X-ray diffractometer (Rigaku, MiniFlex) with Cu Kα radiation. The lattice parameters were calculated by Rietveld analysis using SmartLab Studio II Powder XRD software (Rigaku), and the halide compositions were determined by the lattice parameters. X-ray photoelectron spectroscopy (XPS) measurements were conducted using a photoelectron spectrometer (ULVAC-PHI, PHI X-tool) to analyze the electronic states of the perovskites. Steady-state diffuse reflectance spectra were measured on UV-visible-NIR spectrophotometer (JASCO, V-770). A zoom stereomicroscope (Nikon, SMZ800N) equipped with a microscope camera (Nikon, Digital Sight 1000) was used to image the samples. Scanning electron microscopy (SEM) combined with energy dispersive X-ray spectroscopy (EDS) (JEOL, JCM-7000) was used to analyze the structure and composition of the materials.

### Sample preparation for single-particle photoluminescence (PL) experiments

Cover glasses were purchased from Matsunami-glass, Ltd. and cleaned by sonication in a 20% alkaline detergent solution (AS ONE Corporation, Cleanace) for several hours. The glasses were then washed ten times with distilled and ultrapure water (Milli-Q). Perovskite microcrystals synthesized by using an organic solvent were spin-coated onto the cleaned cover glass as seed crystals. A perovskite-supersaturated HX solution was then dropped onto the perovskite-coated cover glasses to induce crystal growth as the solution cooled. Photoluminescence (PL) measurements were carried out on the grown crystals.

### Single-particle PL measurements

Single-particle PL measurements were conducted using a home-built inverted fluorescence microscope (Nikon, Ti-E) system. During the measurements, we covered the sample holder to prevent solution volatilization and maintained the room temperature at 23 ± 1 °C to preserve dynamic equilibrium. A 405-nm continuous wave (CW) laser (Coherent, OBIS 405LX) or picosecond pulsed laser (Advanced Laser Diode Systems, PiL040X) was used as the excitation source which was focused through an oil-immersion objective lens (Nikon, CFI Plan Apo λ 100× Oil; NA 1.45). The emission from the samples was collected using the same objective lens. A dichroic mirror (Semrock, Di02-R405) and a longpass filter (Semrock, BLP01-458R) were used to block scattered excitation light. Additional fluorescence filters were employed to obtain the appropriate images. An electron-multiplying CCD (EMCCD) camera (Photometrics, Evolve 512) or a color sCMOS camera (Tucsen Photonics, Dhyana 400DC) was used to capture the transmission and PL images. PL spectra were measured using an imaging spectrograph (SOL instruments, MS 3504i) equipped with a CCD camera (Andor, DU416A-LDC-DD). Data were analyzed using ImageJ (https://rsb.info.nih.gov/ij/) and OriginPro 2023 (OriginLab).

### H_2_ production activity tests

A gas chromatograph (Shimadzu, GC-8A) equipped with an MS-5A column and a thermal conductivity detector (TCD) was used to measure the amount of produced H_2_ gas. 10 mg of MAPbBr_*x*_I_3−*x*_ was dispersed in 5 mL of MAPbBr_*x*_I_3−*x*_-saturated HBr/HI solution. The suspensions were purged with Ar gas for 5 min to remove dissolved oxygen, then sealed with a rubber septum. For photocatalytic H_2_ production, the suspension was exposed to visible light (ca. 125 mW·cm^−2^) from a 470 nm LED (Thorlabs, M470L3; nominal wavelength = 470 nm, bandwidth (FWHM) = 25 nm) through a 420 nm sharp cut filter (OptoSigma, SCF-50S-42L). The suspension was vigorously stirred during the experiment at room temperature. The amounts of photogenerated H_2_ gas were measured using a gas chromatograph. During the measurements, we sealed the reactor to prevent solution volatilization and maintained the room temperature at 23 ± 1 °C to preserve dynamic equilibrium.

## Supplementary information

Transparent Peer Review file

Supplementary Information

Description of Additional Supplementary Files

Supplementary Movie 1

Supplementary Data 1

## Data Availability

All data that support the conclusions of this study are available in the paper and the Supplementary Information or from the corresponding author on reasonable request. Source data presented in main manuscript are provided in Supplementary Data 1. The result of microscopic imaging during photodamaging and self-healing processes is provided in Supplementary Movie 1.
