# Supplementary material for: Unassisted self-healing photocatalysts based on Le Chatelier’s principle
Source: Commun Chem. 2025 Apr 14;8:112. doi: 10.1038/s42004-025-01500-7 (PMC11997063; doi:10.1038/s42004-025-01500-7)
Supplement: Supplementary file 2 — Supplementary Information [file 42004_2025_1500_MOESM2_ESM.pdf]

Supplementary Information

Table of Contents

Supplementary Note 1, 2

Supplementary Figures S1–S20

Supplementary Movie 1

Supplementary References

Supplementary Note 1. Tendency of halide anions incorporation

According to the Born equation, the Gibbs free energy change (ΔG) of solvation for an ion can be expressed as follows[1,2]:

$$\Delta G = -\frac{N_A z^2 e_0^2}{8\pi\varepsilon_0 r}(1 - \frac{1}{\varepsilon_s})$$

where N_A is Avogadro's constant, z is the charge of the ion, e_0 is the elementary charge, ε_0 is the permittivity of free space, r is the radius of ion, and ε_s is the dielectric constant of the solvent. In the perovskite-saturated aqueous solution, bromide and iodide anions could undergo solvation by water molecules (i.e., hydration). Considering that each anion has a charge of −1 and ε_s remains constant regardless of the anions, the Born equation primarily depends on the ion radius (r). The ionic radii of bromide and iodide anions are 1.96 Å and 2.20 Å, respectively[3]. Therefore, the smaller bromide anion is more effectively hydrated due to a more negative ΔG. This hydration process results in a greater number of free (unhydrated) iodide anions compared to bromide anions, leading to a preference for iodide anions to form the perovskite structure. Consequently, a deviation from the precursor ratio was observed, as shown in Supplementary Fig. 1a and b.

Supplementary Note 2. Estimation of potential lifetime of photocatalytic reaction system

HX (where X is halide anion) splitting reaction has high impact on various fields such as energy and hygiene industry[5–7]. The self-healing-based (photo)catalytic system offers a strategy for achieving a sustainable society in an environmentally friendly manner. Here, we discuss the lifetime of the photocatalytic reaction system under our experimental conditions. The reaction solution consists of a mixture of HBr, HI, and H_3PO_2 solutions. The concentrations of the original solutions are [HBr] = 8.78 M, [HI] = 7.60 M, and $[H_3PO_2]$ = 9.20 M, respectively. Considering that both HBr and HI are strong acids and fully dissociate in aqueous solution, the pH of the HBr solution is more negative compared to the same volume of the HI solution. To simplify the calculations, we assume the dynamic equilibrium of single-halide $MAPbI_3$ in an HI/H_3PO_2-saturated aqueous solution. In the experiments, 4 mL of HI solution was mixed with 1 mL of H_3PO_2 solution. The concentrations of each component in the reaction mixture are as follows: $[H^+] = [I^-] = 7.60 \times 0.8 = 6.08$ M and $[H_3PO_2] = 9.20 \times 0.2 = 1.84$ M, respectively. Here, the dissociation of H_3PO_2 is not considered due to its relatively high pK_a value (1.23) compared to the pH of this solution (~−0.78). Park et al. demonstrated that $MAPbI_3$ cannot stably maintain its structure when pH > −0.5[4]. Based on these considerations, in our systems, $MAPbI_3$ crystals remain stable as long as $[H^+]$ does not drop below 3.16 (= $10^{0.5}$) M. In other words, the $MAPbI_3$ crystals can stably exist until 14.6 mmol of H^+ are consumed. This value is higher than the total amount of H_3PO_2 in the solution (9.2 mmol). If H_3PO_2 in the solution are depleted, there still remain H^+. Therefore, the minimum lifetime of the (photo)catalytic system can be estimated by dividing 9.2 mmol by the total amount of H_2 generated over 24 h. As shown in Fig. 4c, the highest (photo)catalytic H_2-production activity per ~24 h is 17.3 μmol, meaning that 34.6 μmol of H^+ (= 17.3 μmol × 2) are consumed. Consequently, the estimated lifetime of the reaction system is 9.2 mmol/34.6 μmol ≈ 266 days.

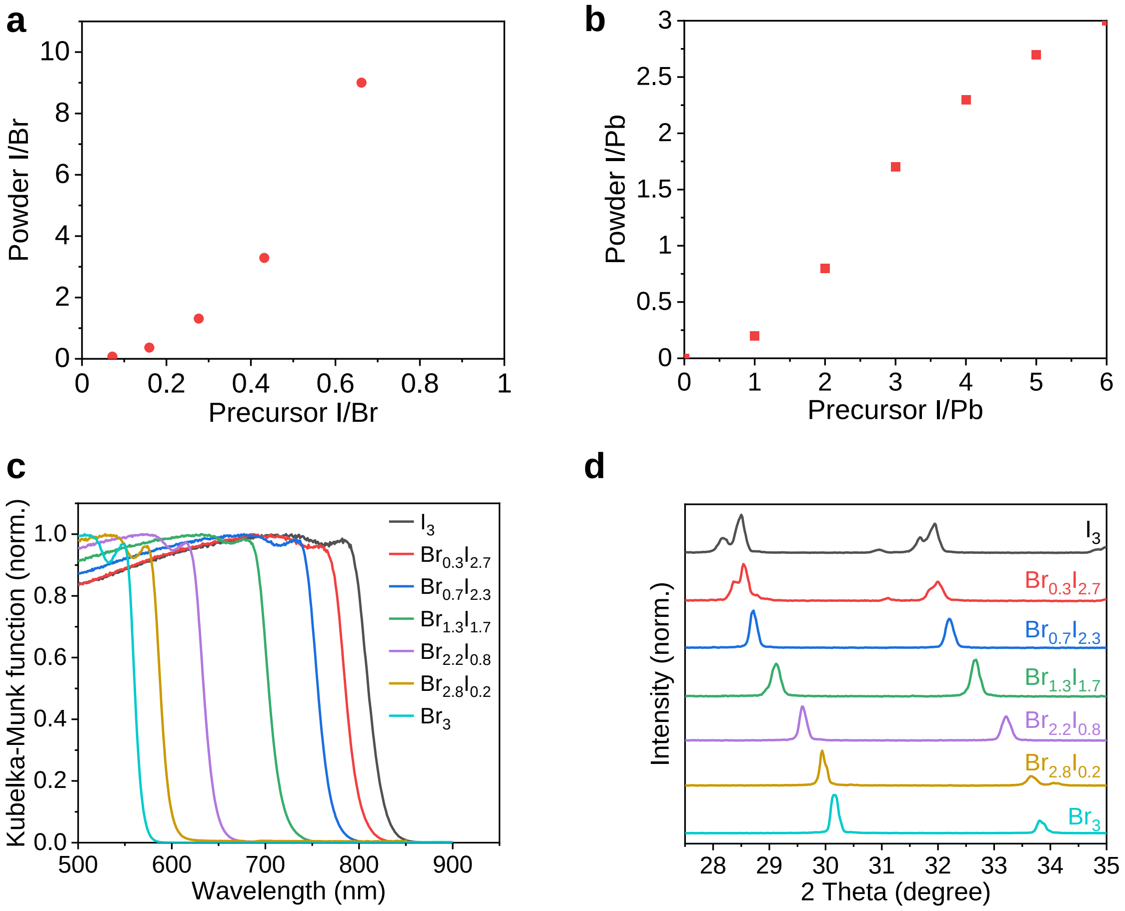

Supplementary Fig. 1 | Characterizations of $MAPbBr_xI_{3-x}$ obtained under dynamic equilibrium conditions. a,b, Correlation between precursor and obtained (**a**) I/Br and (**b**) I/Pb ratios. **c**, Steady-state diffuse reflectance spectra of aqueous synthesized $MAPbBr_xI_{3-x}$ with various halide compositions. **d,** XRD patterns of $MAPbBr_xI_{3-x}$ with various halide compositions.

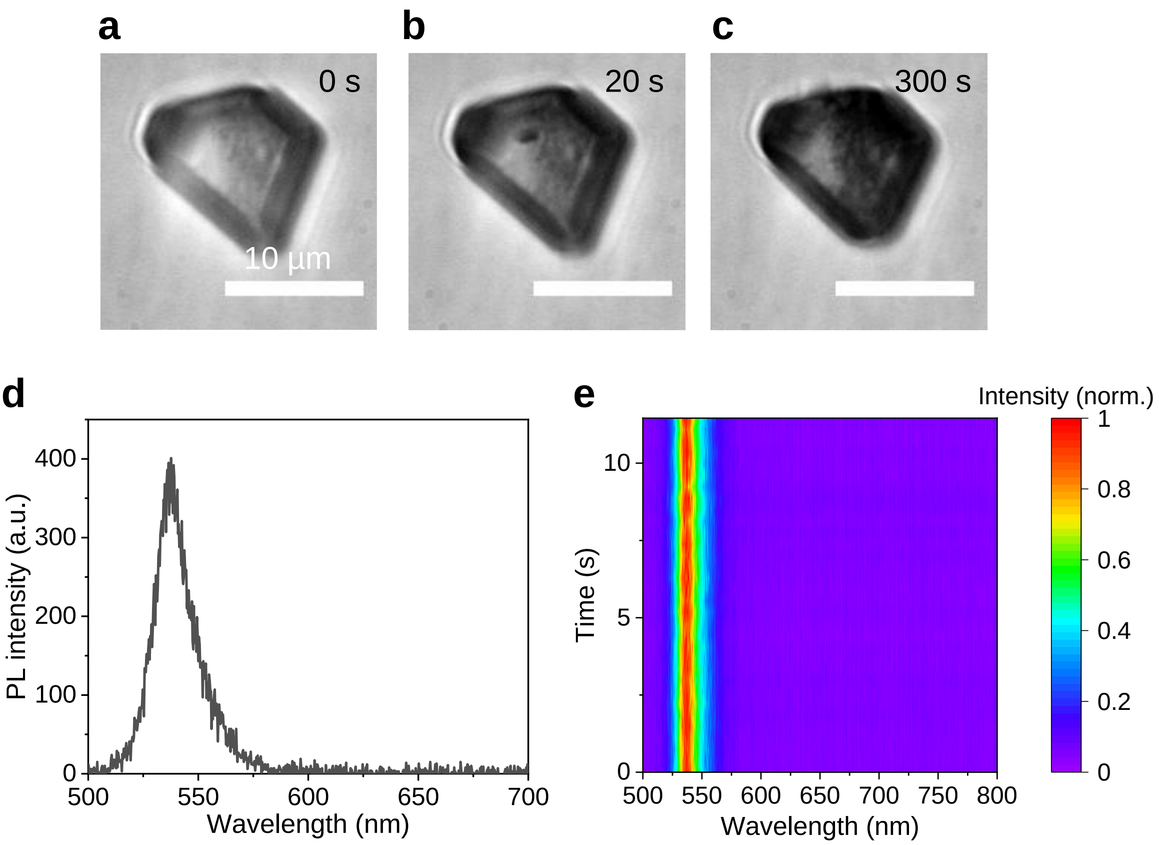

Supplementary Fig. 2 | Characterizations of $MAPbBr_3$ in saturated aqueous solution. a–c, PL and transmission images of $MAPbBr_3$ under photoirradiation. The insets indicate the time after the start of irradiation. A 405-nm CW laser (ca. 1.21 W·cm^{-2}) was used as excitation source. The crystal morphology remained unchanged compared to the mixed-halide $MAPbBr_{2.8}I_{0.2}$, as shown in Fig. 2. **d,e,** PL spectra of $MAPbBr_3$ in aqueous solution. A 405-nm CW laser (ca. 3.18 W·cm^{-2}) was used as excitation source. (**d**) PL spectrum obtained by a single scan and (**e**) time evolution of normalized spectra. The peak wavelength remained unchanged during irradiation.

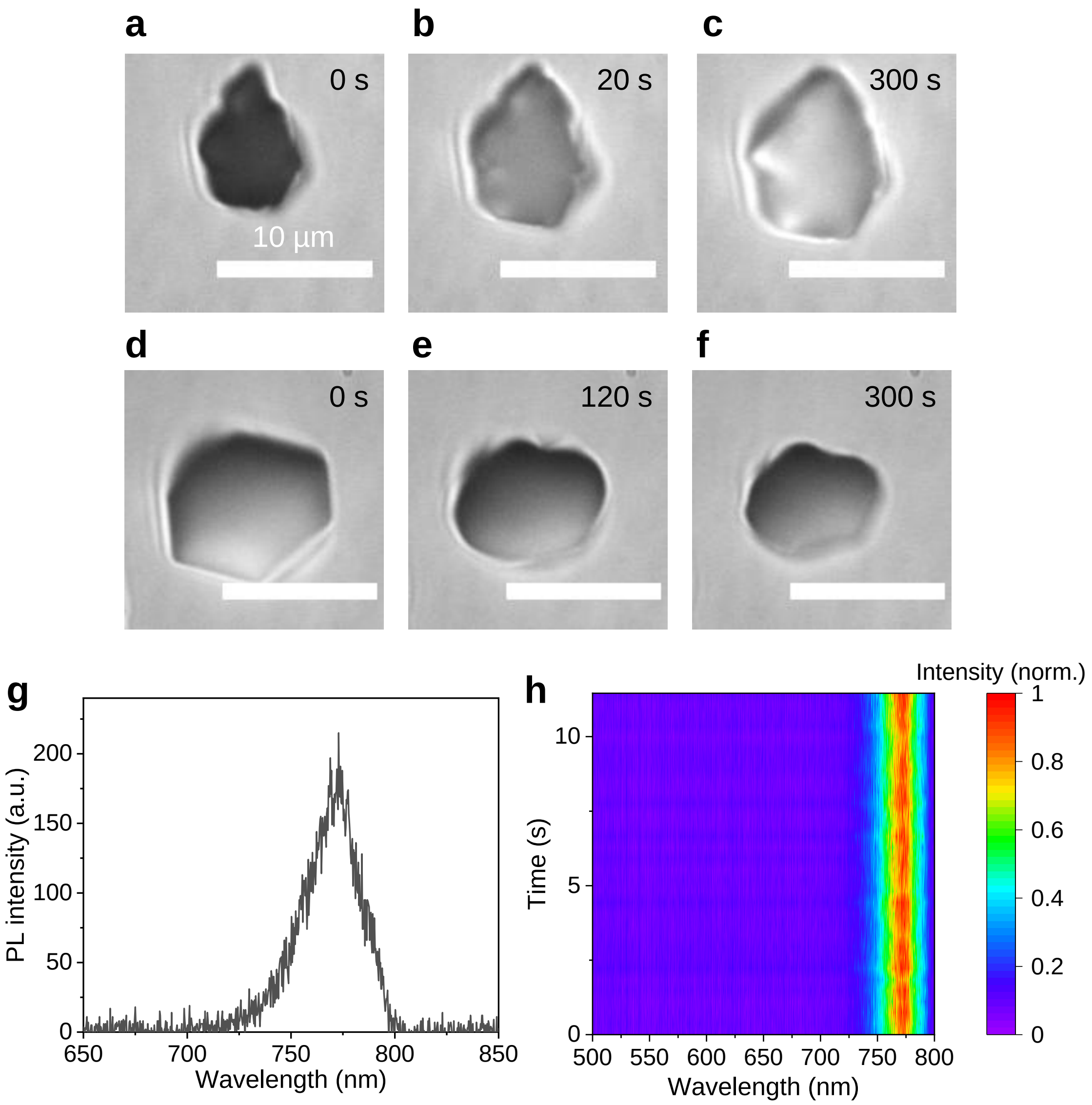

Supplementary Fig. 3 | Characterizations of $MAPbI_3$ in saturated aqueous solution. a–f, PL and transmission images of $MAPbI_3$ under photoirradiation. The insets indicate the time after the start of irradiation. Although the crystal sizes were slightly altered, their morphology remained unchanged compared to the mixed-halide $MAPbBr_{2.8}I_{0.2}$ crystals, as shown in Fig. 2. Panels (**a–c**) and (**d–f**) correspond to observations of the same crystal, respectively. A 405-nm CW laser (ca. 3.18 $W \cdot cm^{-2}$ and 6.07 $W \cdot cm^{-2}$) was used as excitation source to capture the panels (**a–c**) and (**d–f**), respectively. **g,h,** PL spectra of $MAPbI_3$ in aqueous solution. A 405-nm CW laser (ca. 3.18 $W \cdot cm^{-2}$) was used as excitation source. (**g**) PL spectrum obtained by a single scan and (**h**) time evolution of normalized spectra. The peak wavelength remained unchanged during irradiation.

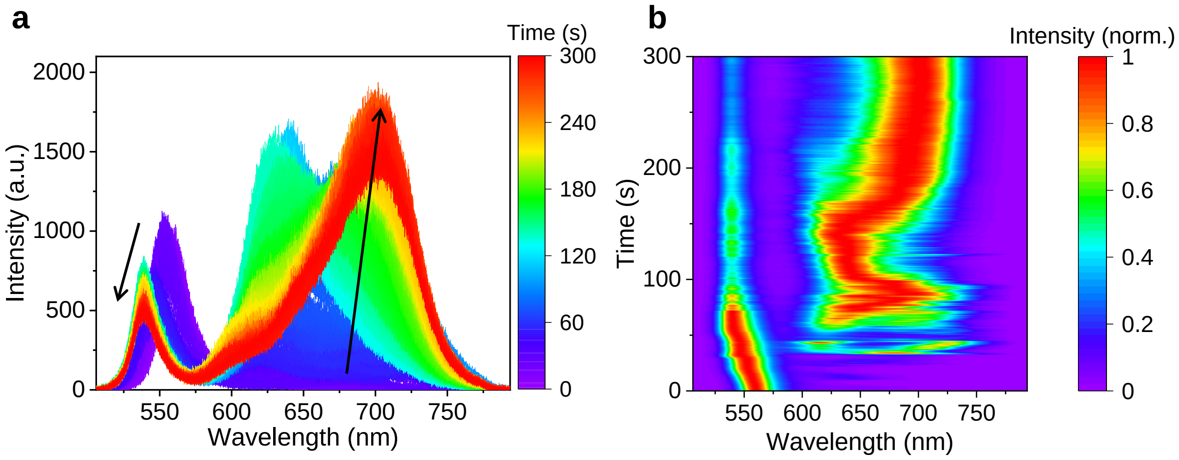

Supplementary Fig. 4 | Characterizations of $MAPbBr_{2.8}I_{0.2}$ in saturated aqueous solution. A 405-nm pulsed laser (ca. 2×10^{-14} J·pulse^{-1}) was used as excitation source. **a,** Time evolution of PL spectra of $MAPbBr_{2.8}I_{0.2}$ under irradiation. **b,** Time evolution of normalized PL spectra. The emission at around 560 nm slightly blue-shifted and weakened, while the emission intensity at around 700 nm increased with increasing irradiation time. This result is consistent with the color imaging of $MAPbBr_{2.8}I_{0.2}$, as shown in Fig. 3. In addition, an intermediate emission state (~650 nm) is observed when the measurement time is between 120 s and 180 s. Light-induced halide phase segregation progresses as the light irradiation time increases. The emission wavelength of perovskites reflects their halide composition. Therefore, this intermediate emissive state can be attributed to transient, partially phase-segregated perovskites [8,9].

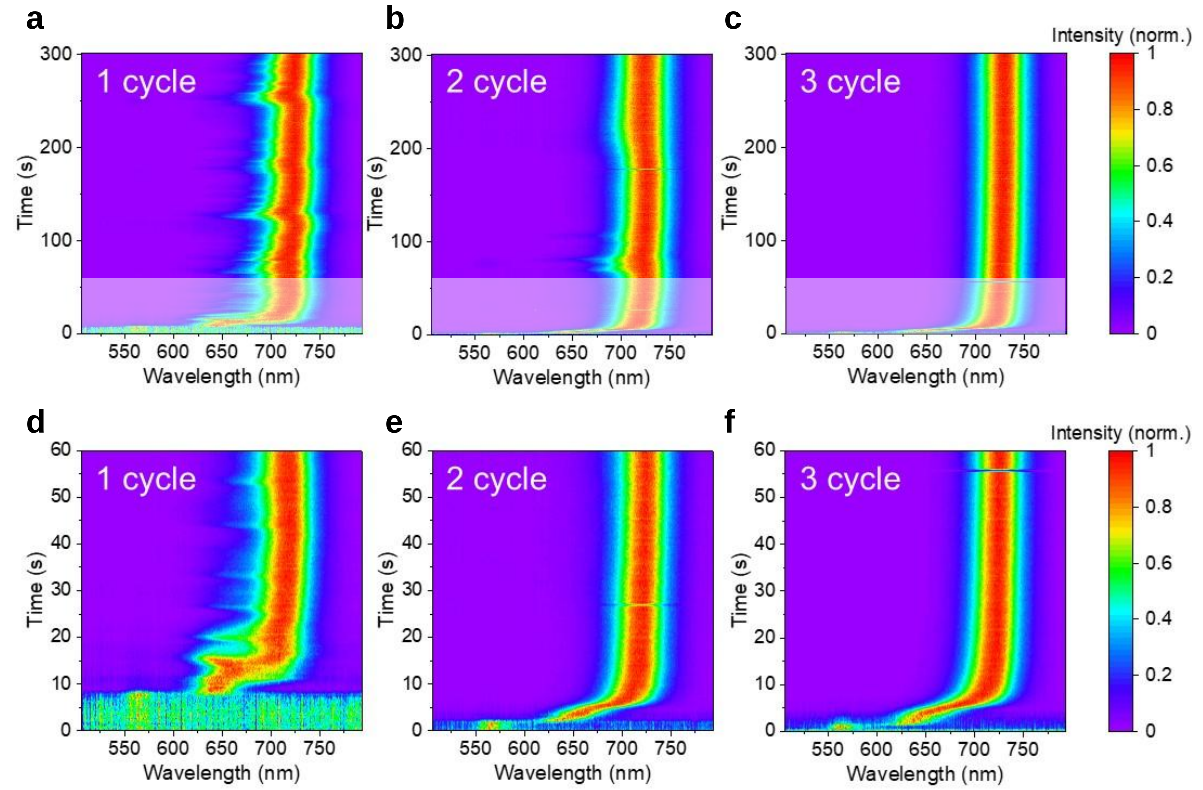

Supplementary Fig. 5 | PL properties of $MAPbBr_{2.8}I_{0.2}$ in aqueous solution. a–c, Time evolution of normalized PL spectra. A 405-nm pulsed laser (ca. 2×10^{-14} J·pulse^{-1}) was used as excitation source. The peak wavelength remained nearly constant in each repeating cycle. **d–f,** Magnified spectra in the 1–60 s region of each cycle. In the first cycle, the detected intensity was quite weak compared to the second and third cycles. This can be explained by light-induced defect passivation, also known as photoactivation[10–12].

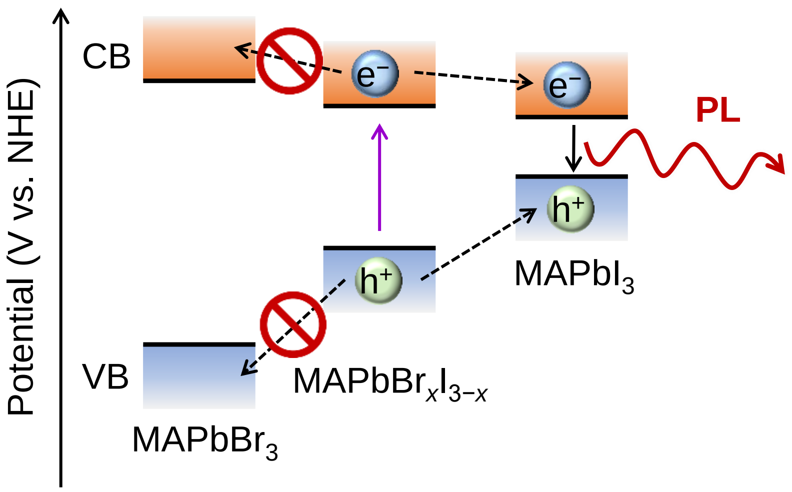

Supplementary Fig. 6 | Schematic band alignment of $MAPbBr_3$, $MAPbI_3$, and $MAPbBr_xI_{3-x}$. Photogenerated charge carriers anisotropically transfer to iodide-rich domains due to their narrower band gap[13]. CB and VB represent conduction band and valence band, respectively.

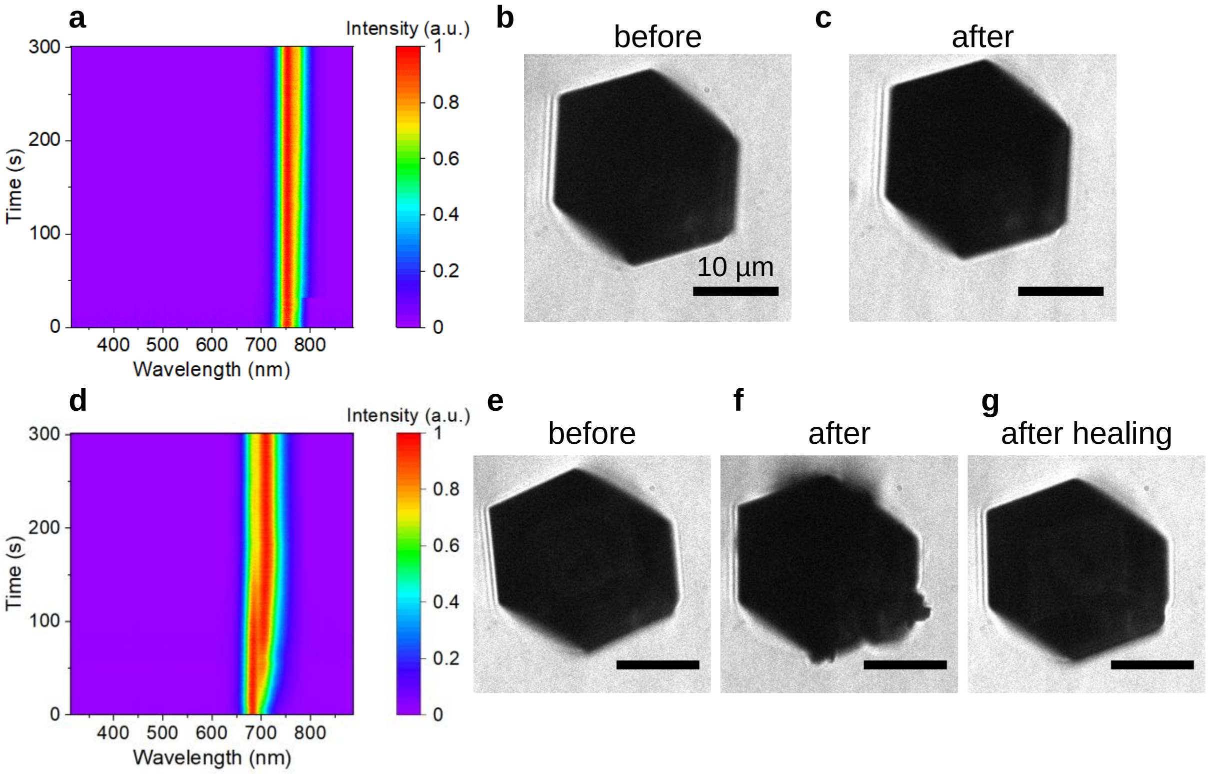

Supplementary Fig. 7 | Characterizations of $MAPbBr_{0.3}I_{2.7}$ (a–c) and $MAPbBr_{1.3}I_{1.7}$ (d–g). A 405-nm CW laser (ca. 3.18 $W \cdot cm^{-2}$) was used as excitation source. (**a**) Time evolution of normalized PL spectra of $MAPbBr_{0.3}I_{2.7}$. No significant change in the spectral shape was observed during irradiation. (**b,c**) Morphology imaging of the crystal before and after photoirradiation. Their morphology remained unchanged after photoirradiation. (**d**) Time evolution of normalized PL spectra of $MAPbBr_{1.3}I_{1.7}$. The PL spectra show slightly red-shifted PL as irradiation time increased. (**e–g**) Morphology imaging of the crystal before and after photoirradiation, and after self-healing for 1 h. The crystal was damaged after irradiation and exhibited self-healing behavior under dark conditions. The threshold halide composition for phase segregation has been reported as $MAPbBr_{0.6}I_{2.4}$[14]. If the ratio of bromide anions falls below 0.6, phase segregation does not occur, which can be explained by thermodynamic stability. This explains why $MAPbBr_{0.3}I_{2.7}$ did not exhibit phase segregation behavior.

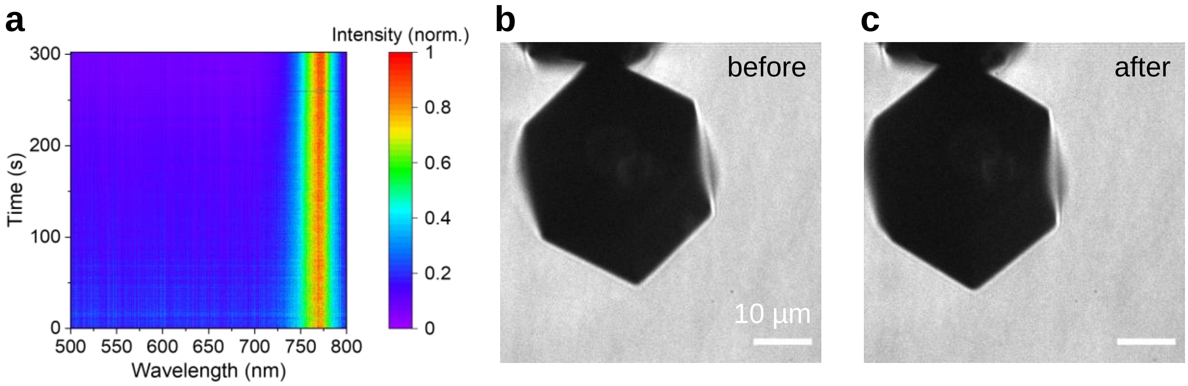

Supplementary Fig. 8 | Characterizations of $MAPbI_3$ with a small amount of bromide in aqueous solution. A 405-nm CW laser (ca. 9.44 $W{\cdot}cm^{-2}$) was used as excitation source. **a,** Time evolution of normalized PL spectra. The peak wavelength remained nearly the same in each repeating cycle. **b,c,** Morphology imaging of the crystal before and after photoirradiation. The crystal morphology remained unchanged compared to the mixed-halide $MAPbBr_{2.8}I_{0.2}$, as shown in Fig. 2. As laser irradiation time increased, the PL intensity increased, a phenomenon referred to as photo-activation or light-curing. In $MAPbI_3$, photo-activation is attributed to the annihilation of Frenkel defects under photoirradiation[15]. Additionally, it has been reported that the defect formation energy of $MAPbBr_3$ is higher than that of $MAPbI_3$[16], indicating that the defect density in $MAPbBr_3$ is lower than in $MAPbI_3$. Thus, significant photo-activation was not observed in $MAPbBr_3$, as shown in Supplementary Fig. 9.

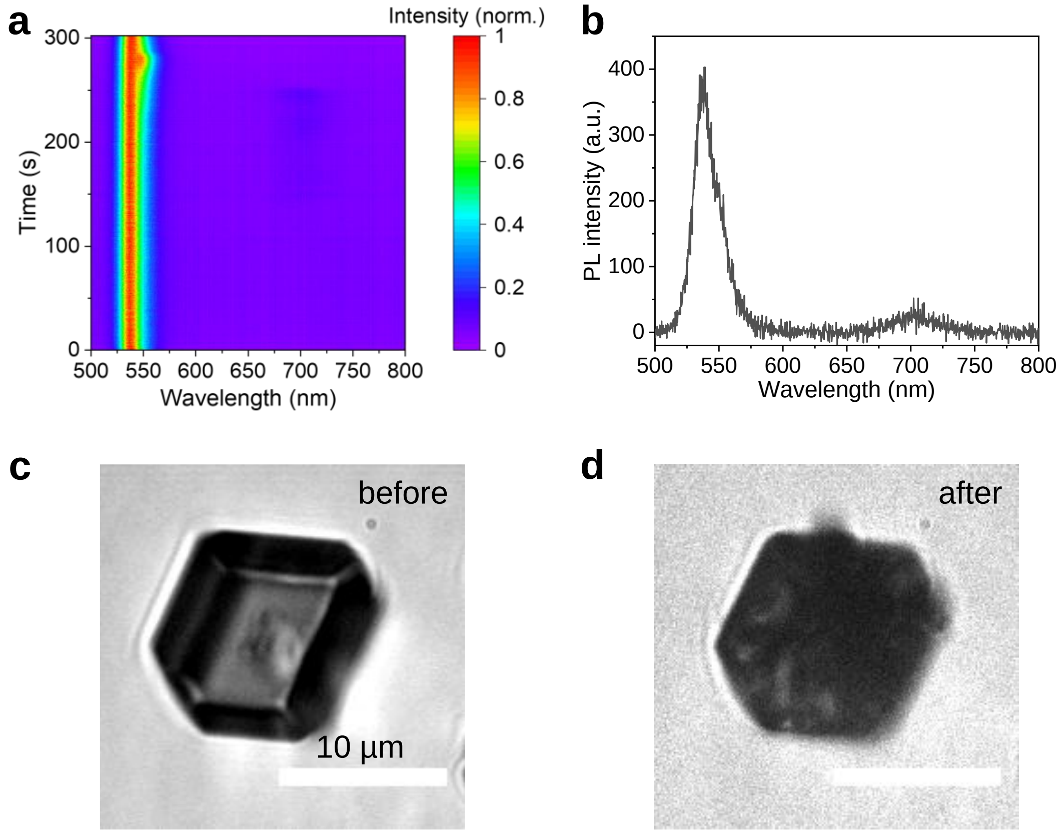

Supplementary Fig. 9 | Characterizations of $MAPbBr_3$ with a small amount of iodide in aqueous solution. a, Time evolution of normalized PL spectra. The peak wavelength remained nearly the same in each repeating cycle. A 405-nm CW laser (ca. 9.44 $W \cdot cm^{-2}$) was used as excitation source. **b,** Sliced PL spectrum at 241 s in the panel (**a**). **c,d,** Morphology imaging of the crystal before and after photoirradiation. A 405-nm CW laser (ca. 3.18 $W \cdot cm^{-2}$) was used as excitation source. The crystal morphology exhibited a damaged state after irradiation, similar to the mixed-halide $MAPbBr_{2.8}I_{0.2}$. The reason why PL from bromide perovskites did not decrease is that crystal destruction primarily occurs in iodide-rich domains, which emit ~700 nm PL, while bromide-rich domains remain relatively intact, as shown in Supplementary Fig. 11.

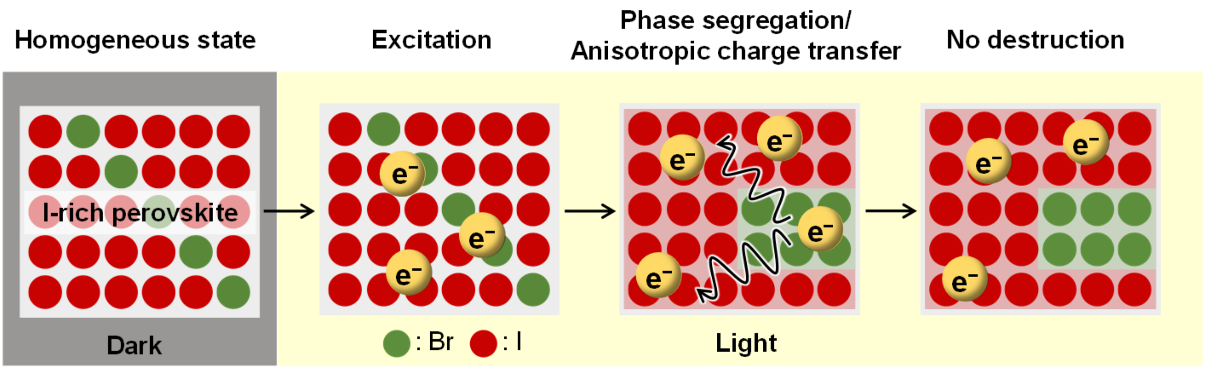

Supplementary Fig. 10 | Schematic illustration of the reason why $MAPbI_3$ which contains small amount of bromide crystal was not damaged (destructed). No crystal destruction was observed possibly due to the lower carrier concentrations caused by charge diffusion throughout the crystal.

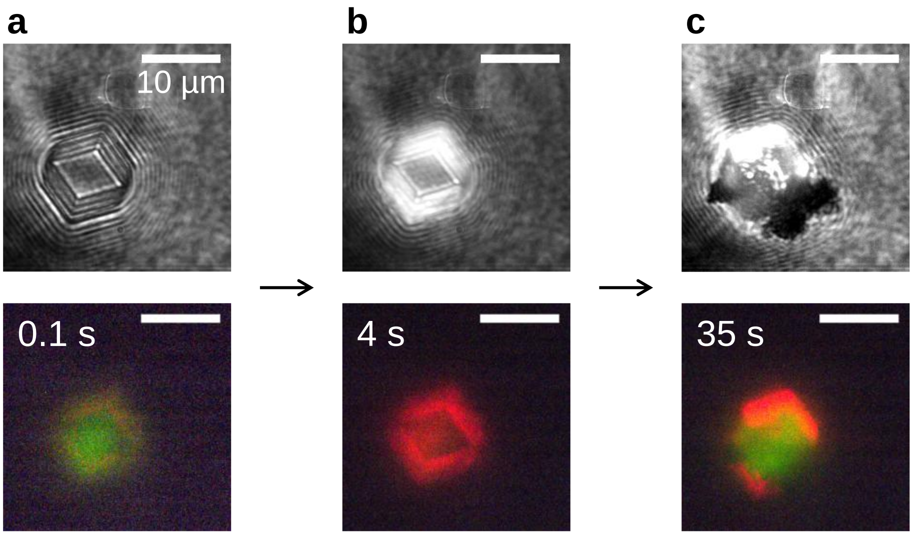

Supplementary Fig. 11 | Simultaneous observation of PL and transmission images of $MAPbBr_{2.8}I_{0.2}$. This measurement was conducted using the same setup as shown in Fig. 2, but with an 810 nm LED instead of room light to monitor the morphological changes. A 405-nm CW laser (ca. 747 mW·cm^{-2}) was used as excitation source. The PL and transmission images were captured using a color sCMOS camera and an EMCCD camera, respectively. In our microscopic experiments, we observe PL only from the near-surface region (~120 nm) due to the limited penetration depth of light. Immediately after starting light irradiation, the crystal emits weak green PL (**a**). As we continued photoirradiation, the crystal emits red PL due to light-induced phase segregation (**b**). When the damaging reaction initiates, the regions near the damage emit green PL because the iodide-rich domains are no longer present (**c**). The processing timescales of phase segregation and crystal destruction differ, allowing us to observe and analyze their behavior separately using single-particle spectroscopy.

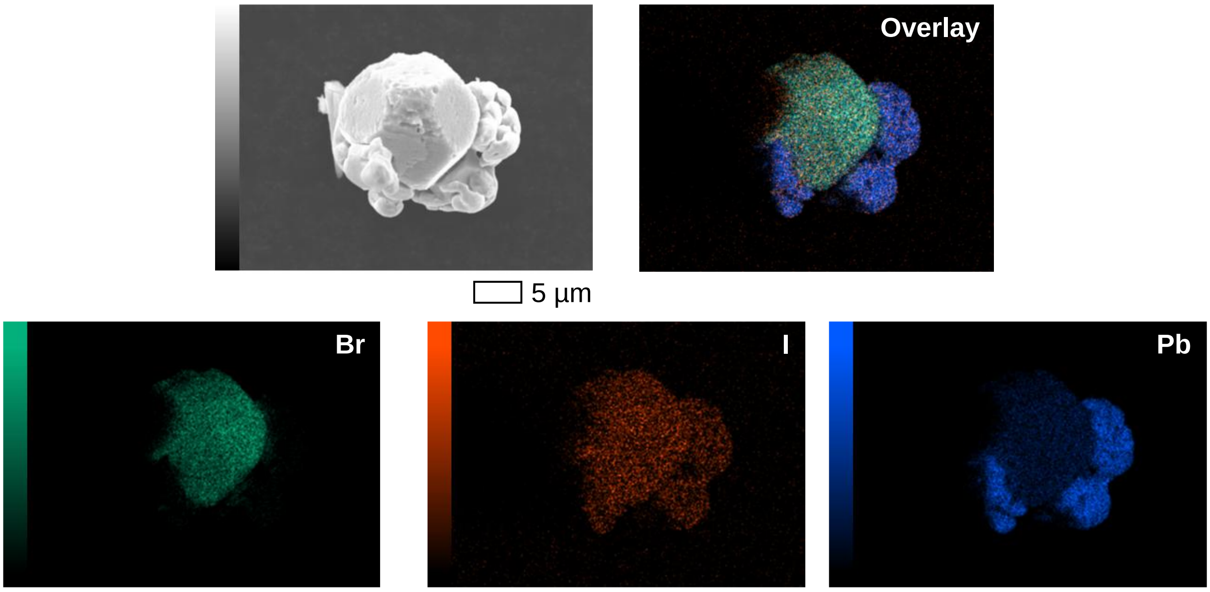

Supplementary Fig. 12 | SEM-EDS images of $MAPbBr_{2.8}I_{0.2}$ after photoirradiation. Each color represents an element: green for Br, red for I, and blue for Pb. After irradiation, Pb-rich domains were observed, indicating that photo-induced metallic Pb formation occurred.

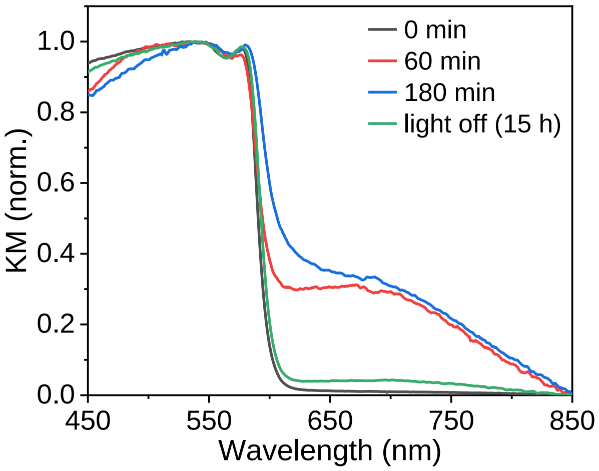

Supplementary Fig. 13 | Diffuse-reflectance spectra of $MAPbBr_{2.8}I_{0.2}$. Each spectrum was normalized within the range of 0 to 1. Photoirradiation induces a broad band in the lower energy region, which can be mainly attributed to the absorption of photogenerated metallic Pb. After the sample was kept for 15 h under dark conditions, the intensity of this band significantly decreased, suggesting the progression of self-healing reactions.

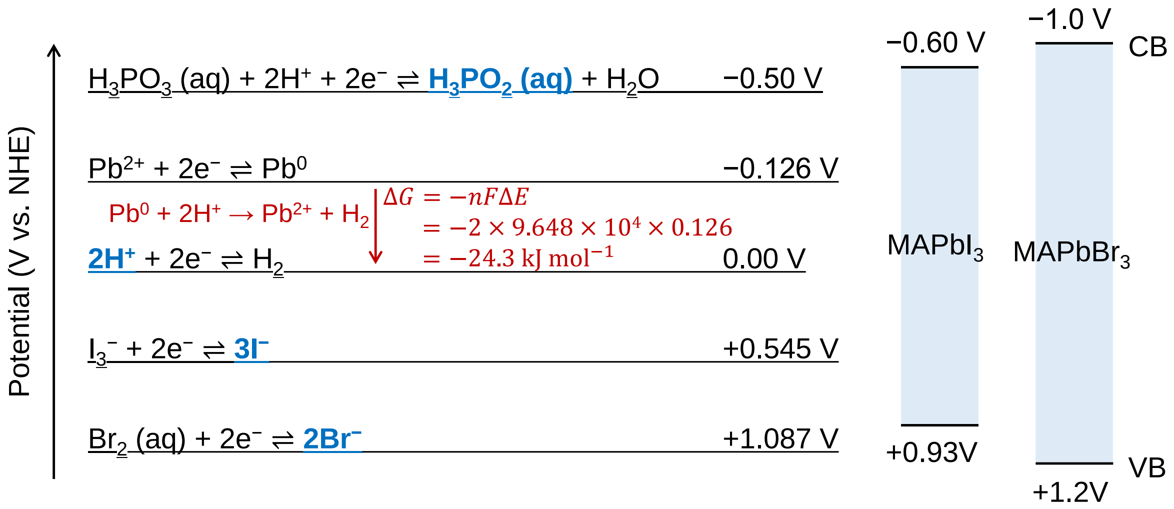

Supplementary Fig. 14 | Potential alignment of related half reactions in perovskite-saturated aqueous solution and band edge energies of $MAPbBr_3$ and $MAPbI_3$[17,18]. The chemicals shown in blue represent the dominant existing species in each reaction. Once metallic Pb^0 is generated, it spontaneously oxidizes to Pb^{2+} with a $\Delta G < 0$, due to the abundance of protons in the solution.

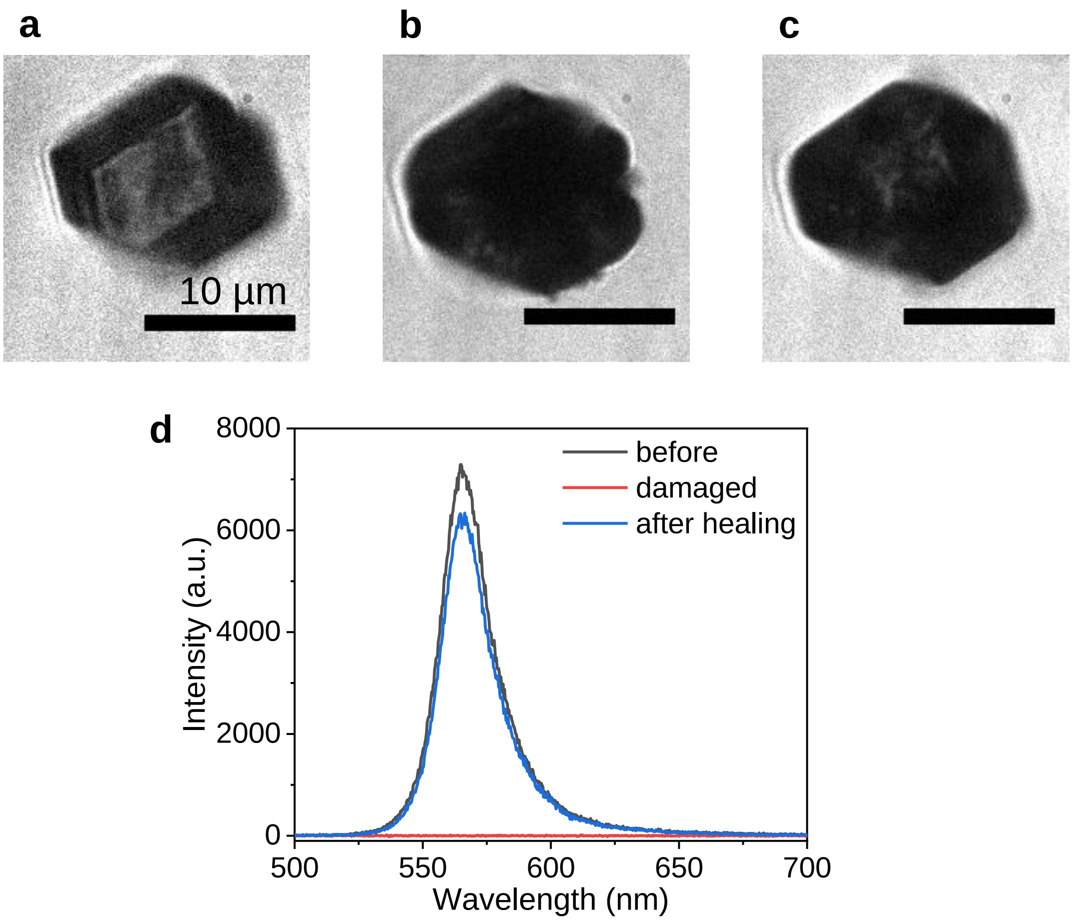

Supplementary Fig. 15 | Characterizations of $MAPbBr_{2.8}I_{0.2}$ before photodamaging, after photodamaging, and after self-healing reactions. a–c, Transmission images of samples: (**a**) before photodamaging, (**b**) after photodamaging, and (**c**) after self-healing reactions. **d**, Photoluminescence spectra of each sample. A 405-nm pulsed laser (ca. 3×10^{-12} J·pulse^{-1}) was used as the excitation source.

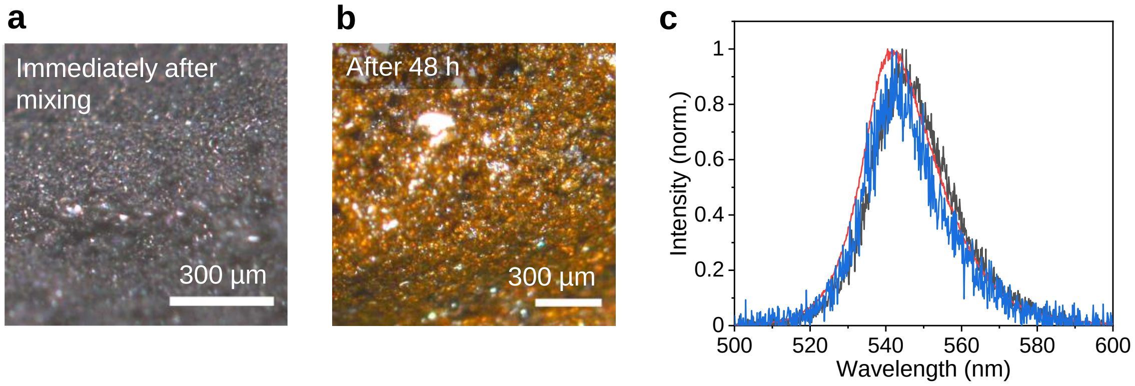

Supplementary Fig. 16 | Characterizations of $MAPbBr_3$ obtained from metallic Pb^0 powders in aqueous solution. a,b, Optical images of Pb^0 powders (**a**) immediately after mixing with the solution and (**b**) 48 hours after mixing. **c,** PL spectra of the orange-colored regions (see the panel **b**) A 405-nm CW laser (ca. 747 $mW \cdot cm^{-2}$) was used as excitation source. Regarding $MAPbBr_3$ synthesis from Pb powder, we can highlight a few key observations. First, stirring the reaction solution accelerates the formation of $MAPbBr_3$. Additionally, once the surface of the Pb powder is covered by the generated orange-colored $MAPbBr_3$, the oxidation of Pb^0 to Pb^{2+} is inhibited, as evidenced by the formation of hydrogen bubbles, as shown in Eq. (2) in the main text. Therefore, we assume that several factors are closely related to self-healing reactions, specifically ensuring that the seed (core) remains intact and maintaining good contact between the seed (core) and the precursor solution.

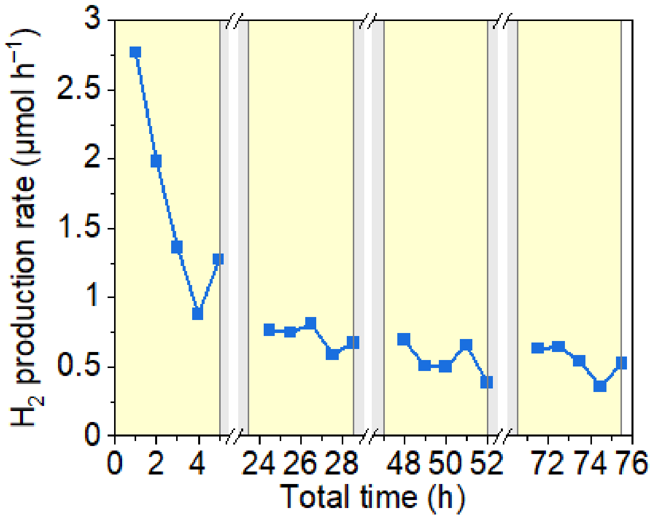

Supplementary Fig. 17 | Hydrogen-production rates of $MAPbBr_{2.8}I_{0.2}$. The yellow-shaded regions represent the periods during which the excitation light was irradiated, while the gray-shaded regions indicate the times when the perovskites were kept in darkness. The production rates were calculated from Fig. 4c.

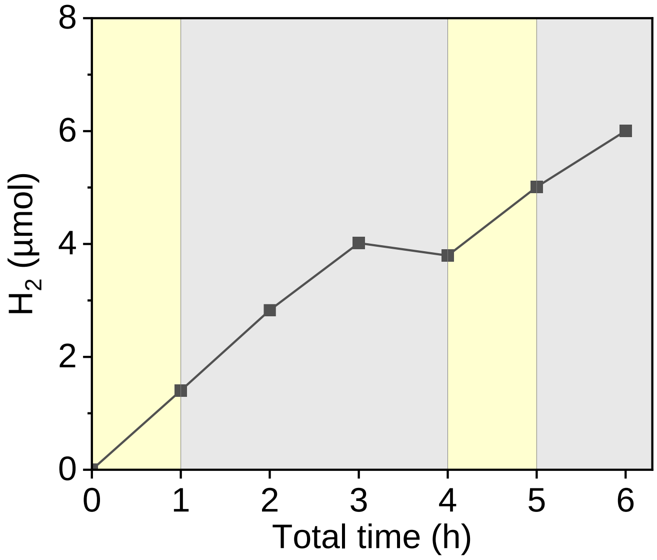

Supplementary Fig. 18 | (Photo)catalytic hydrogen-production activity of $MAPbBr_{2.8}I_{0.2}$. The yellow-shaded regions represent the periods during which the excitation light was irradiated, while the gray-shaded regions indicate the times when the perovskites were kept in darkness.

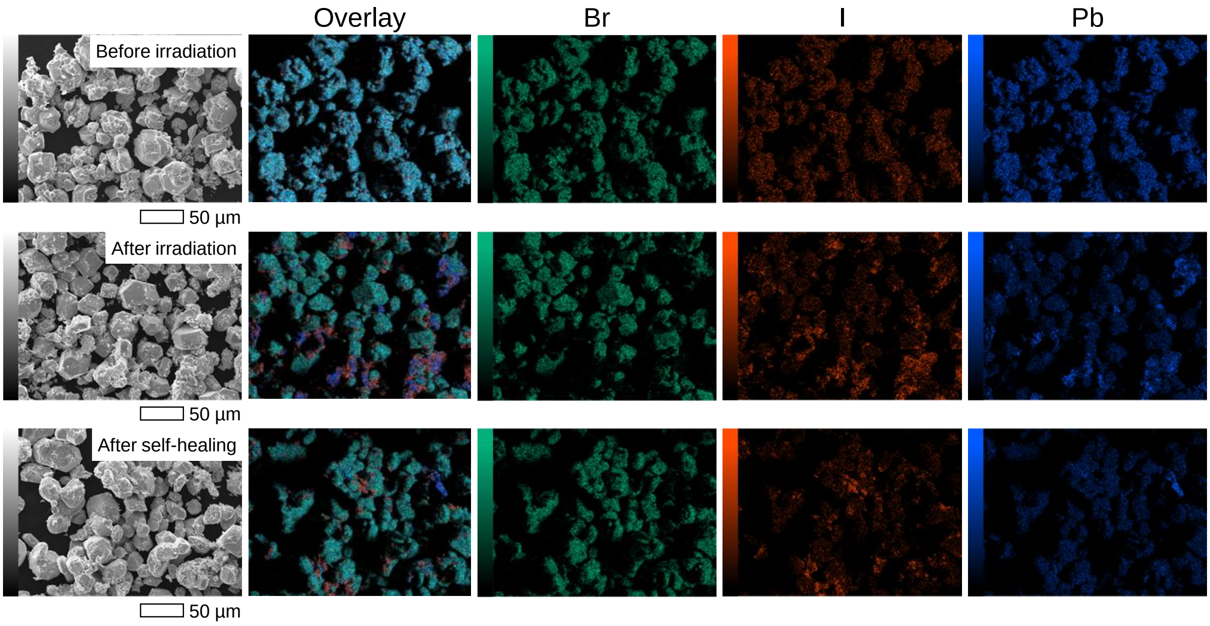

Supplementary Fig. 19 | SEM-EDS images of $MAPbBr_{2.8}I_{0.2}$ obtained under various conditions. The samples were irradiated for 5 h in an aqueous solution, followed by 18.5 h of storage in the solution under dark conditions to induce self-healing reactions. This procedure is the same as that used in the photocatalytic activity tests.

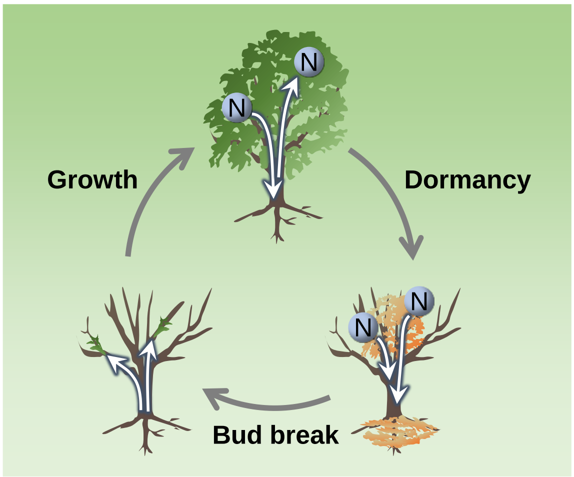

Supplementary Fig. 20 | Schematic illustration of the deciduous tree cycle. Energy is stored in the stem when the leaves are shed. The stored energy is then utilized for the growth of the next generation of leaves.

Supplementary Movie 1. Damaging and self-healing reactions of $MAPbBr_{2.8}I_{0.2}$ under photoirradiation in aqueous solution. This movie (frame rate = 200 fps) was captured using a home-built fluorescence microscope. The scale bar is 10 µm. The damaging reaction was observed under 405-nm CW laser irradiation (ca. 780 $mW \cdot cm^{-2}$) and the self-healing reaction proceeded after stopping the laser irradiation. The images were captured at a frame rate of 5 fps.

Supplementary References

[1] Born, M. Volumen und hydratationswärme der ionen. *Z. Physik* **1**, 45–48 (1920).

[2] Silva, G. M., Maribo-Mogensen, B., Liang, X. & Kontogeorgis, G. M. Improving the Born equation: origin of the Born radius and introducing dielectric saturation effects. *Fluid Phase Equilib.* **576**, 113955 (2024).

[3] Marcus, Y. Ionic radii in aqueous solutions. *Chem. Rev.* **88**, 1475–1498 (1988).

[4] Park, S. et al. Photocatalytic hydrogen generation from hydriodic acid using methylammonium lead iodide in dynamic equilibrium with aqueous solution. *Nat. Energy* **2**, 16185 (2017).

[5] Yeo, R. S. and Chin, D.-T. A hydrogen-bromine cell for energy storage applications. *J. Electrochem. Soc.* **127**, 549 (1980).

[6] Taylor, G. R. & Butler, M. A comparison of the virucidal properties of chlorine, chlorine dioxide, bromine chloride and iodine. *J. Hyg.* **89**, 321–328 (1982).

[7] Huskinson, B., Rugolo, J., Mondal, S. K. & Aziz, M. J. A high power density, high efficiency hydrogen–chlorine regenerative fuel cell with a low precious metal content catalyst. *Energy Environ. Sci.* **5**, 8690–8698 (2012).

[8] Draguta, S. et al. Rationalizing the light-induced phase separation of mixed halide organic-inorganic perovskites. *Nat. Commun.* **8**, 200 (2017).

[9] Motti, S. G. et al. Phase segregation in mixed-halide perovskites affects charge-carrier dynamics while preserving mobility. *Nat. Commun.* **12**, 6955 (2021).

[10] Mosconi, E., Meggiolaro, D., Snaith, H. J., Stranks, S. D. & De Angelis, F. Light-induced annihilation of Frenkel defects in organo-lead halide perovskites. *Energy Environ. Sci.* **9**, 3180–3187 (2016).

[11] Tian, Y. et al. Enhanced organo-metal halide perovskite photoluminescence from nanosized defect-free crystallites and emitting sites. *J. Phys. Chem. Lett.* **6**, 4171–4177 (2015).

[12] Cordero, S., Carson, P., Estabrook, R., Strouse, G. & Buratto, S. Photo-activated luminescence of CdSe quantum dot monolayers. *J. Phys. Chem. B* **104**, 12137–12142 (2020).

[13] Jang, D. M. et al. Reversible halide exchange reaction of organometal trihalide perovskite colloidal nanocrystals for full-range band gap tuning. *Nano Lett.* **15**, 5191 (2015).

[14] Hutter, E. M. et al. Thermodynamic stabilization of mixed-halide perovskites against phase segregation. *Cell Rep. Phys. Sci.* **1**, 100120 (2020).

[15] Mosconi, E., Meggiolaro, D., Snaith, H. J., Stranks, S. D. & De Angelis, F. Light-induced annihilation of Frenkel defects in organo-lead halide perovskites. *Energy Environ. Sci.* **9**, 3180–3187 (2016).

[16] McGovern, L., Futscher, M. H., Muscarella, L. A. & Ehrler, B. Recent progress in halide perovskite nanocrystals for photocatalytic hydrogen evolution. *J. Phys. Chem. Lett.* **11**, 106 (2020).

[17] deBethune, A. J., Licht, T. S. & Swendeman, N. The temperature coefficients of electrode potentials: the isothermal and thermal coefficients—the standard ionic entropy of electrochemical transport of the hydrogen ion. *J. Electrochem. Soc.* **106**, 616–625 (1959).

[18] Zhang, Z. et al. Recent progress in halide perovskite nanocrystals for photocatalytic hydrogen evolution. *Nanomaterials* **13**, 106 (2023).
