# Supplementary material for: Unassisted self-healing photocatalysts based on Le Chatelier’s principle
Source: Commun Chem. 2025 Apr 14;8:112. doi: 10.1038/s42004-025-01500-7 (PMC11997063; doi:10.1038/s42004-025-01500-7)
Supplement: Supplementary file 3 — Description of Additional Supplementary Files [file 42004_2025_1500_MOESM3_ESM.pdf]

Description of Additional Supplementary Files

File name: Supplementary Movie 1

Description: Damaging and self-healing reactions of $MAPbBr_{2.8}I_{0.2}$ under photoirradiation in aqueous solution. This movie (frame rate = 200 fps) was captured using a home-built fluorescence microscope. The scale bar is 10 µm. The damaging reaction was observed under 405-nm CW laser irradiation (ca. 780 $mW \cdot cm^{-2}$) and the self-healing reaction proceeded after stopping the laser irradiation. The images were captured at a frame rate of 5 fps.

File name: Supplementary Data 1

Description: Source Data
